# Supplementary material for: Secondary care consultant clinicians’ experiences of conducting emergency care and treatment planning conversations in England: an interview-based analysis
Source: BMJ Open. 2020 Jan 20;10(1):e031633. doi: 10.1136/bmjopen-2019-031633 (PMC7044868; doi:10.1136/bmjopen-2019-031633)
Supplement: Author's manuscript [file bmjopen-2019-031633.draft_revisions.pdf]

BMJ Open is committed to open peer review. As part of this commitment we make the peer review history of every article we publish publicly available.

When an article is published we post the peer reviewers' comments and the authors' responses online. We also post the versions of the paper that were used during peer review. These are the versions that the peer review comments apply to.

The versions of the paper that follow are the versions that were submitted during the peer review process. They are not the versions of record or the final published versions. They should not be cited or distributed as the published version of this manuscript.

BMJ Open is an open access journal and the full, final, typeset and author-corrected version of record of the manuscript is available on our site with no access controls, subscription charges or pay-per-view fees (http://bmjopen.bmj.com).

If you have any questions on BMJ Open's open peer review process please email info.bmjopen@bmj.com

BMJ Open

# Secondary Care Clinicians' Experiences of Conducting Emergency and Advance Care Planning Conversations: An Interview-Based Analysis



| | |
|---|---|
| Journal: | *BMJ Open* |
| Manuscript ID | bmjopen-2019-031633 |
| Article Type: | Research |
| Date Submitted by the Author: | 15-May-2019 |





| | |
|---|---|
| Complete List of Authors: | Eli, Karin; University of Warwick, Division of Health Sciences, Warwick Medical School<br>Ochieng, Cynthia; Cardiff University, School of Medicine<br>Hawkes, Claire; University of Warwick Warwick Medical School, Clinical Trials Unit<br>Perkins, Gavin; University of Warwick, Clinical Trials Unit<br>Couper, Keith; University of Warwick, Warwick Medical School<br>Griffiths, Frances; University of Warwick, Warwick Medical School<br>Slowther, Anne-Marie; University of Warwick, Warwick Medical School |





| | |
|---|---|
| Keywords: | Emergency care treatment planning, Advance care planning, Clinicians' experiences, Do not attempt cardiopulmonary resuscitation (DNACPR), QUALITATIVE RESEARCH, Decision making |
| | |



SCHOLARONE™
Manuscripts

# Secondary Care Clinicians' Experiences of Conducting Emergency and Advance Care Planning Conversations: An Interview-Based Analysis

Karin Eli[1], Cynthia Ochieng[2], Claire Hawkes[1], Gavin D. Perkins[1, 3], Keith Couper[1, 3], Frances Griffiths[1]*, Anne-Marie Slowther[1]*

[1] Warwick Medical School, University of Warwick
[2] School of Medicine, Cardiff University
[3] Critical Care Unit, University Hospitals Birmingham NHS Foundation Trust, Birmingham

*These authors share senior authorship

Corresponding author: Karin Eli (Karin.Eli@warwick.ac.uk)

For peer review only

## Abstract

Objective: To examine secondary-care clinicians' experiences of conducting conversations about treatment escalation with patients and their relatives, using the Recommended Summary Plan for Emergency Care and Treatment (ReSPECT) process.

Design: Semi-structured interviews following ward round observations.

Setting: Two National Health Service hospitals in England.

Participants: Fifteen medical and surgical consultants from 10 specialties, observed in 14 wards.

Analysis: Interview transcripts were analysed using thematic analysis.

Results: Three themes were developed: (1) Determining when and with whom to conduct a ReSPECT conversation; (2) Framing the ReSPECT conversation to manage emotions and relationships; and (3) Reaching ReSPECT decisions. The results showed that when timing ReSPECT conversations, clinicians rely on their predictions of a patient's short-term prognosis; when framing ReSPECT conversations, clinicians seek to minimize distress and maximize rapport; and when involving a patient or a patient's relatives in decision-making discussions, clinicians are guided by their level of certainty about the patient's illness trajectory.

Conclusions: The management of uncertainty about prognoses and about patients' emotional reactions is central to secondary-care clinicians' experiences of timing and conducting ReSPECT conversations.

## Keywords

Emergency care treatment planning; advance care planning; clinicians' experiences; critical care; do not attempt cardiopulmonary resuscitation (DNACPR); decision making; qualitative research; recommended summary plan for emergency care and treatment (ReSPECT); uncertainty.

## Article summary: strengths and limitations of this study

- Data were collected from clinicians from ten medical specialities, thereby representing diverse secondary-care environments and clinical attitudes to emergency and advance care planning.



For peer review only - http://bmjopen.bmj.com/site/about/guidelines.xhtml

- Each clinician was first shadowed during a ward round and then interviewed. This allowed for an in-depth discussion of ReSPECT conversations in relation to cases observed by the researcher, thus grounding the interviews in specific and varied case examples.
- The findings reported in this paper are limited by the study's focus on consultants' interviews. In the two study sites, consultants were responsible for signing ReSPECT forms; however, as other members of multidisciplinary teams also participate in ReSPECT conversations, including their perspectives and experiences would have been valuable.
- The interviews took place within the first year of ReSPECT implementation in the two study sites. Thus, it is possible that some findings reflect experiences related to early implementation.

For peer review only



For peer review only - http://bmjopen.bmj.com/site/about/guidelines.xhtml

## Introduction

Do not attempt cardiopulmonary resuscitation (DNACPR) decisions allow clinical practitioners to withhold cardiopulmonary resuscitation when cardiac arrest occurs as part of a person's natural and irreversible dying process (Rabkin et al., 1976). DNACPR decisions are made when CPR cannot realistically succeed, when the burdens that resuscitation would pose to the patient outweigh potential benefits, or when a patient requests not to be resuscitated (Resuscitation Council UK, 2014; General Medical Council, 2010). While DNACPR guidelines are clearly articulated, several studies and reviews have found that, in practice, DNACPR processes are fraught with ambiguity. Clinicians have varying, sometimes-divergent understandings of DNACPR decision-making processes and the ethics thereof, leading to inconsistencies in how decisions are made, implemented, and recorded (Perkins, 2016; Mockford et al., 2015; Freeman et al., 2015; Clements et al., 2014). These inconsistencies may lead to lower quality of care; indeed, some clinicians misinterpret DNACPR decisions as limiting other aspects of treatment, while others administer CPR inappropriately, failing to follow patients' wishes for the withholding of resuscitation (Perkins, 2016; Fritz et al., 2010; Findlay et al., 2012; Cohn et al., 2013; McAdam et al., 2005). Notably, clinicians often communicate poorly about DNACPR with patients and their relatives, and some are reluctant to discuss resuscitation, thereby excluding patients from the decision-making process (Perkins, 2016; Mockford et al., 2015; Myint et al., 2006; Myint et al., 2010; Fritz et al., 2014).

This paper is part of a larger study, funded by the National Institute for Health Research (NIHR), which evaluates the Recommended Summary Plan for Emergency Care and Treatment (ReSPECT) (NIHR HS&DR, 2016). Launched in 2017 across NHS Trusts, ReSPECT is an emergency care treatment plan (ECTP) developed in response to the gaps observed in the DNACPR process. ReSPECT builds on research conducted in the US, the UK, and Canada, which found that programmes that integrate DNACPR with advance care planning increase clarity about trajectories of care and reduce harm to patients (Fritz et al., 2017). As an ECTP, ReSPECT emphasizes patient involvement in the decision-making process and places resuscitation within a wider treatment context, focusing not only on the withholding of treatment, but also on identifying which treatments will be offered.

The ReSPECT process is designed to guide clinicians and patients in discussing emergency care treatment plans, with the ReSPECT form supporting the ReSPECT process and recording a summary of the discussion and its outcomes. The ReSPECT form and its associated guidance documents were developed in 2016 by the ReSPECT working group. Chaired by the Resuscitation Council (UK) and Royal College of Nursing, the ReSPECT working group had representation from patients, professional organisations (Royal Colleges, British Medical Association) regulatory bodies (General Medical Council, Nursing Midwifery Council), the Care Quality Commission, NHS organisations (Acute, Community and Ambulance Trusts) and patient and public members. The ReSPECT form is distinct from DNACPR forms, recording a wider discussion of patients' values and the range of treatments that should or should not be considered in future emergency situations, including, but not limited to, cardiopulmonary resuscitation. The completed ReSPECT form is patient-centred: it records patients' wishes and is kept in the patients' possession, allowing them to communicate the treatment plans recorded in the form when they move from one healthcare setting to another.

In the present paper, we report findings from interviews with secondary-care clinicians, exploring decision-making about the ReSPECT process in two NHS organisations that have recently



For peer review only - http://bmjopen.bmj.com/site/about/guidelines.xhtml

implemented ReSPECT. Our aim is to inform future implementation plans across the NHS and to provide focus to further qualitative research on how ReSPECT becomes integrated into health professionals' practice.

**Methods**

Fifteen consultants (six women and nine men) from two acute NHS teaching hospitals in England were interviewed from August to December 2017 as part of a wider ongoing study, aimed at evaluating the implementation of the ReSPECT process. Together, the 15 consultants represented 14 wards and 10 medical specialities. In Site 1, interviews took place 7-10 months after ReSPECT had been implemented; in Site 2, interviews took place 11-12 after ReSPECT had been implemented. Prior to implementation of ReSPECT the hospitals used the Resuscitation Council (UK) Do Not Attempt Cardiopulmonary Resuscitation (DNACPR) system to record advance decisions relating to Resuscitation. We observed no differences related to ReSPECT implementation timelines between the two sites.

Consultants were approached for participation by their local site principal investigator or research nurse. All participants provided written informed consent prior to taking part in the study. The study's research fellow, a public health researcher, shadowed each consultant during a ward round, to observe when and how consultants engaged in ReSPECT conversations with their patients. The researcher interviewed each consultant following the observation, typically within a day or two. The interviews were semi-structured and included questions about three cases observed during the ward round, to explore the consultant's decision-making about holding a ReSPECT conversation in each case, alongside questions about wider experiences with ReSPECT. This two-stage design, whereby each clinician was first shadowed during a ward round and then interviewed, grounded the interviews in specific and varied case examples. The interviews lasted from 15 to 53 minutes, with a median time of 37 minutes, and were digitally recorded and transcribed.

The interview transcripts were analysed by the study's senior research fellow (SRF), a medical anthropologist, using thematic analysis (Braun and Clarke, 2006). First, the SRF read the interview transcripts to identify initial codes. The transcripts were then coded closely, with most codes developed at the level of sentences or sentence clauses. The SRF reviewed the coded interviews, and grouped the codes to develop themes. The themes were continuously revised throughout the process of reviewing the coded interviews, leading to 16 emerging themes, which were grouped into overarching themes. To ensure intercoder reliability, four of the 15 interviews were analysed independently by another SRF, a health services researcher. The two SRFs discussed the codes, identified differences and potential disagreements, and discussed these until they reached consensus. In total, five initial overarching themes were developed: three focused on the ReSPECT conversation, one focused on clinicians' value judgments, and one focused on the ReSPECT form. After they achieved consensus, the SRFs discussed the five overarching themes with two of the senior co-authors, clinicians with research expertise in medical ethics and medical sociology. Together, they decided to focus the analysis on the three overarching themes concerned with the ReSPECT conversation. Finally, the first SRF reviewed all interview transcripts to ensure the three themes represented the data accurately. Throughout the analytic process, coding was conducted



For peer review only - http://bmjopen.bmj.com/site/about/guidelines.xhtml

using word processing software. To maintain participant confidentiality, the gender-neutral pronouns they/their are used to refer to all clinicians.

Patient and public involvement (PPI)

The study has been supported by a PPI group, members of which provided feedback on a draft of the manuscript. They agreed with the manuscript's findings and offered suggestions for areas to investigate further in our future research, in light of their own experiences as patients or carers.

**Results**

*Theme 1: Determining when and with whom to conduct a ReSPECT conversation: Uncertainty management and catalysts for discussion*

Given time constraints, clinicians had to determine which patients were most in need of a ReSPECT conversation, and when this conversation should be conducted. Making these determinations was fraught with uncertainty. To manage this uncertainty, clinicians relied on their predictions and imaginings of patients' immediate futures, using the ward round to piece together prognostic puzzles. For example, explaining why he did not initiate a ReSPECT conversation with a patient in her 90s, this clinician said:

> *she's otherwise recovering well (…) I thought the chances of her, as it were, needing any of the interventions you might discuss on a ReSPECT form were small. (Site 1, C10)*

The key to initiating a ReSPECT conversation, this clinician later explained, was predicting a trajectory of deterioration:

> *It's the deteriorating patients, patients with end-of-life conditions for whatever reason, be it cancer or organ failure and any patient where they might suddenly deteriorate. (Site 1, C10)*

Another clinician said they used the ward round to predict whether a patient was likely to experience a 'catastrophe'; such a prediction, they said, would warrant initiating a ReSPECT conversation:

> *Particularly where you're seeing acutely ill patients and you're seeing them for the first time as, as an acute physician, I think the prompt is how likely you think it is that this patient may have a catastrophe, may have a cardiac arrest, may have a sudden severe deterioration. (Site 1, C11)*

This clinician, like numerous others in the sample, linked the focus on predicted deterioration to the time constraints of the ward round. As another clinician explained:

> *I think in the context of a post-take ward round where I am time limited I prioritise those patients for whom these conversations are most likely to be required for this admission. So it may well be that ReSPECT conversations were appropriate for more of the patients that I saw today in terms of potentially being last year of life. But they were not decisions that were required today. (Site 2, C17)*



For peer review only - http://bmjopen.bmj.com/site/about/guidelines.xhtml

While time constraints were frequently cited, they were not the only factor underlying clinicians' focus on predicted deterioration. Explaining why they were less likely to initiate a ReSPECT conversation with some patients, this clinician positioned their decision-making as culturally embedded:

> *I think for the time being the culture is still the ReSPECT form is mainly for when people deteriorate. I think sometimes asking people a hypothetical question when they're really quite well, it's difficult to frame it. (Site 1, C09)*

This clinician linked their focus on deterioration as the primary prompt for a ReSPECT conversation to the difficulty of asking patients to imagine a hypothetical catastrophic scenario. Imagining difficult scenarios, however, was central to ReSPECT conversations. Since initiating ReSPECT conversations depended on clinicians' predictions of patients' short-term prognoses, ReSPECT conversations engaged patients with clinicians' predictive thinking. This process was often challenging, as patients did not necessarily share in the logics and concepts of time posed by their clinicians:

> *[P]eople find, "What if?" challenging. So if I say, "What if you're going to deteriorate? We need to make a decision what we would do about ITU." A lot of patients and relatives will hear about us saying, "You're deteriorating, you're going to need ITU." They don't hear the "What if?" (Site 2, C17)*

Like others, this clinician explained that while they framed possible future scenarios in subjunctive – that is, potential or conditional – terms, patients and relatives tended to understand these in definitive future terms. Another clinician explained that, rather than joining a dialogue on potential scenarios, some patients and relatives expressed distress over what they understood as a terminal prognosis:

> *…even though I've said "I am going to let you go home now, have you thought about what you would like in the future?", and then they say "why am I going to die? You're telling me I am going to die aren't you!" (Site 1, C01)*

In addition to predicted deterioration, clinicians timed ReSPECT conversations according to calculations of risk related to a wider network of actors, including patients, patients' relatives, and other clinicians. For example, some clinicians said they were reluctant to hold ReSPECT conversations with pre-operative patients, as these discussions could bias surgeons or demoralise patients:

> *[The patient] was going to undergo an operation and I feel very uncomfortable discussing resuscitation just before the operation (…) if he does not want to be resuscitated, it influences the surgeon to some extent. (Site 2, C14)*

> *…we don't often talk about ReSPECT form because it's, you know, when you deteriorate. And in some ways with elective surgery they're very much focused on consenting for surgery and talking about rehab after surgery rather than deterioration from surgery. (Site 1, C08)*



For peer review only - http://bmjopen.bmj.com/site/about/guidelines.xhtml

Elsewhere in the interview, this surgeon said the best time to initiate a ReSPECT conversation was immediately after surgery – a particularly opportune time because, while risk of complications was higher after surgery, patients' distress was likely to be lower, and relatives were likely to be present:

> *…often, often there's a family around at that point because it's usually an emergency admission and the family come in a day or two later. So you can involve the parties that you need to at that point in time. It's a relatively rare event for a patient to die on the operating table, if they're going to, if they're going to succumb it's usually over the following few days. (Site 1, C08)*

The presence of relatives was a prompt for ReSPECT conversations, not least because relatives are essential to conducting the ReSPECT process with patients who lack capacity. In this example, a clinician discussed a case where a visit from a patient's relatives prompted a ReSPECT conversation:

> *[H]is family were there so I took the opportunity while they were all there to express not only that he was perhaps more unwell than they had recognised, and that he was getting better with treatment, and to explore what their feelings were about escalation of care, particularly whether intensive care would be appropriate for him. (Site 2, C17)*

This clinician considered the presence of relatives crucial in timing the ReSPECT conversation due to uncertainty about escalating the patient's care. Other clinicians, however, spoke of the presence of relatives as important for finalizing and communicating a medical decision, rather than deliberating about a trajectory of care.

*Theme 2: Conducting the ReSPECT conversation: Managing emotions and relationships*

Most ReSPECT conversations implicated a triad of patient, clinician(s), and relative(s). For patients who lacked capacity, this triad was essential to the ReSPECT process, with relatives or other advocates called upon to speak on the patient's behalf. However, while patients with capacity could speak privately with their clinicians, they often involved their relatives, framing the ReSPECT conversation and their own decision-making as familial. In these cases, clinicians clarified they included relatives in the conversation, but did not involve them in decision-making:

> *…you've had a discussion, the patient says, "Look, I don't want anything done, doctor," I think it's still very valuable to the next of kin to know that. (…) but we're not asking the family to participate in the discussion if the patient has already made their wishes clear in a reliable way. (Site 1, C11)*

Although clinicians tended to describe relatives' involvement as valuable, they also described it as potentially problematic, characterising family members as either compliant and 'sensible', or as non-compliant and 'difficult'. According to some clinicians, relatives sometimes challenged clinical decisions – specifically, decisions against resuscitation – because they misunderstood what 'not for resuscitation' meant for the patient's future care:

> *Sometimes you have relatives who are very emotional, sometimes they think when you say 'not for resuscitation' means you're going to stop all treatment. (Site 2, C16)*



For peer review only - http://bmjopen.bmj.com/site/about/guidelines.xhtml

In other cases, clinicians said relatives misunderstood their role in the ReSPECT conversation as that of 'decision-maker', worrying about how a 'not for resuscitation' decision might reflect on them:

> *Often what happens is the relatives feel that you're asking them to make the decision… and again because they've been misled by the media, they feel that if they say, yes, make them not for resuscitation, that they might be seen as a money grabbing. (Site 2, C12)*

Disagreement between clinicians and patients' relatives could carry consequences for patient care, particularly when relatives who held legal power of attorney (LPA) attempted to overturn a clinical decision. In those cases, clinicians advocated for their clinical decision, taking the role of acting on the patient's behalf:

> *I try to explain to them that by keeping them alive, you are, you are, you are prolonging their agony. (…) I try to avoid confrontation with them (…) But sometimes we have to, when I can see clearly that there is going to be harm, then I have to, even if they have the LPA. (Site 2, C14)*

Another relational aspect of the ReSPECT conversation was the consideration of other, sometimes absent, clinicians. Several clinicians spoke about the importance of identifying the 'right' clinician to conduct a ReSPECT conversation – often, the consultant or the GP regularly charged with the patient's care. In post-take ward rounds, some clinicians avoided conducting ReSPECT conversations with patients who were usually seen by their colleagues. In this example, a clinician explained why they chose not to complete a ReSPECT form with a patient who had a localised infection:

> *I could've completed a ReSPECT form but I didn't because I, effectively I'm not looking at her responsible consultant. (Site 2, C12)*

Later in the interview, this clinician explained that the patient's condition did not warrant an urgent ReSPECT conversation. Given the lack of urgency, they deferred to the patient's 'usual physicians, who obviously know her prognosis'. It would be inappropriate, this clinician argued, to conduct a ReSPECT conversation with a patient whose consultants evidently did not deem it necessary.

Clinicians deferred ReSPECT conversations until the 'right' clinicians could conduct them not only because patients' usual consultants were more knowledgeable about these patients' medical histories, but also because these consultants had established rapport with the patients. In this example, a clinician explained why they chose to conduct a ReSPECT conversation but leave the final decision for a future discussion between the patient and her usual consultant. The patient, this clinician explained, was not 'receptive' to an earlier ReSPECT conversation with her usual consultant. As such, this clinician viewed their role as providing a second opinion to support the consultant's, rather than as finalizing a ReSPECT decision.

> *…I didn't feel as if I was going to be welcomed to take that further with her myself. So I thought it was better than to say, to see her back to her normal consultant then the next time. (Site 2, C13)*



For peer review only - http://bmjopen.bmj.com/site/about/guidelines.xhtml

For similar reasons, other clinicians suggested that ReSPECT conversations were best conducted in primary care settings, led by patients' GPs rather than by clinicians they first met during an acute care admission. In response to the researcher's question, 'So you think this is something that should be discussed in the community?', this clinician said:

> *Definitely because I think it makes… patients feel less vulnerable… when they are in hospital they feel vulnerable plus they don't know us (…) they might have known the GP or have some sort of on-going or community matron or something that's a bit more of a long term relationship. (Site 1, C04)*

The importance clinicians placed on rapport was closely connected to their concerns over trustworthiness. Worries about being perceived as untrustworthy led some clinicians to avoid or delay ReSPECT conversations with some patients. As described by clinicians, ReSPECT conversations, if not framed carefully, could undermine the process of building trust with patients.

> *One of my worries is that patients, if you're not careful with your language, a patient might interpret a discussion about what to do in the event of deterioration, escalation, CPR, etcetera, as you giving up on them, as you not being prepared to do everything that you can to get them over their illness. (Site 1, C11)*

The timing of ReSPECT conversations could also affect trust building. This clinician, for example, suggested that initiating a ReSPECT conversation too early would shake the patient's trust:

> *… you want to make sure you still have the rapport with the patient, that they see you as somebody that's there to help them (…) and if you feel that the patient is not quite ready to talk about it or they don't want to then if you kind of push it they'll see you negatively. (Site 1, C04)*

Another clinician, a surgeon, described a case where they conducted a ReSPECT conversation before operating on a seriously ill patient. While the ReSPECT conversation was carefully timed from a medical perspective, it forced the patient to confront difficult scenarios that destabilized her trust in the surgeon:

> *So she doesn't want to talk about whether or not she's going to die on the operating table, or whether or not she's going to get her post-op chest infection or a lung embolus or whatever else could happen. But that process makes us talk about it at that point in time. (…) as soon as you mention that sentence about what would you like to do and if things were taking a turn for the worse (…) she's switched, she's completely switched off. (Site 1, C08)*

Notably, clinicians were concerned about being perceived as trustworthy because they identified the ReSPECT conversation as a catalyst for potential distress for patients and relatives. To manage the difficult emotions that often arose during ReSPECT conversations, clinicians used various techniques: from avoiding the conversation if the patient was expected to react aggressively or become overwhelmed, to initiating a series of conversations to ease patients and relatives into their future



For peer review only - http://bmjopen.bmj.com/site/about/guidelines.xhtml

trajectory. For example, one clinician deferred ReSPECT conversations with patients recently diagnosed with terminal cancer to avoid overwhelming them:

> *I've generally just told them they've got incurable cancer and it, to go on straight from that to a ReSPECT conversation is too much. But I will say that it exists and that it may be something they want to consider and then ask somebody else to follow it up. (Site 2, C17)*

Alongside concerns over patients' emotional wellbeing, several clinicians said that previous experiences with patients or relatives who became upset made them cautious about initiating and framing ReSPECT conversations. One clinician, who explained that 'we worry about the angry and anxious one[s]' (Site 2, C17), described beginning each ReSPECT conversation by framing it as common and routine, to pre-empt patients' upset reactions. Another clinician, who described ReSPECT conversations as 'emotionally very draining', conducted repeated ReSPECT conversations to manage relatives' distress:

> *So if you can get some background knowledge, and if they are so in shock that they can't take anything in then it's okay to come back another time. (…) I would prefer to sit away in a, in a room together with a nurse accompanying me, so that you've got a bit of time to yourself and make sure that they know you've got time to listen to them and questions and things. (Site 1, C09).*

The availability of sufficient time and adequate space influenced clinicians' capacity to conduct ReSPECT conversations. Many conversations, of necessity, took place during ward rounds, and the crowdedness, urgent pace, and lack of privacy in acute wards limited clinicians' ability to conduct the in-depth ReSPECT conversations they envisioned as appropriate. This clinician, for example, argued that ReSPECT conversations necessitated the quiet environment of the patient's home or GP surgery:

> *…this is quite a serious and significant discussion that should not take place in a very busy, busy place. It should either happen when the patient is comfortable in their own home, or, or they have gone to see their, gone to see their GP… (Site 2, C14)*

Hectic ward environments, in this clinician's experience, implicated an urgency and sensory onslaught that, together with patients' acute conditions, led to compromised conversations. The lack of sufficient time to conduct ReSPECT conversations in acute care wards was a pervasive concern across the sample:

> *…it takes time and it sort of stirs up emotions both in you and in the patient (…) so it can be very difficult, mmm, not least because you want to do it well and yet you know we were on a ward round which isn't an ideal kind of, ideally you'd come back and spend 20 minutes with each of them wouldn't you and their families and talk to them at some length. (Site 1, C06)*

Notably, this clinician suggested that lack of time was not simply a logistical issue, but a factor that reduced the ability to conduct careful ReSPECT conversations and manage the emotions that arose during ReSPECT conversations.



For peer review only - http://bmjopen.bmj.com/site/about/guidelines.xhtml

*Theme 3: Reaching ReSPECT decisions: Involving versus informing*

The extent to which ReSPECT conversations engaged with patients' wishes depended on clinicians' clarity or uncertainty about patients' trajectories. When clinicians had clear predictions for patients' short-term prognoses, they tended to lead ReSPECT conversations, taking an informative and persuasive stance. For example, when asked by the researcher, 'Are there times when you find yourself pushing the discussion in a particular way?', this clinician responded:

> *Yes, I think if you genuinely feel that it would be completely futile and that you would only be prolonging an unpleasant death then yes, you do, you do tend to push the discussion in one way or another. (Site 1, C10)*

Clinicians often used words such as 'futile', 'frail', or 'co-morbid' when describing cases in which they took a persuasive stance. Futility, as clinicians framed it, foreclosed discussion of patient preferences. The conversation focused on patient preferences only when clinicians were uncertain about a patient's trajectory:

> *I think that the times where it's very important to discuss with a patient whether they would be appropriate for resuscitation is if it's a patient that maybe is potentially a candidate for intensive care, Level 3 care, that isn't so frail and co-morbid that we feel it would be utterly futile. (Site 2, C12)*

Because they approached ReSPECT conversations according to perceptions of prognostic clarity and uncertainty, many clinicians described the ReSPECT conversations in which they typically engaged – conversations with patients at imminent risk – as processes of navigation and persuasion. For example, one clinician described handling a patient's son's concerns by 'steer[ing]' the conversation:

> *I went in with quite clear views of what had to be done and as you say the patient's son started to suggest that 'actually he would want to be resuscitated wouldn't you Dad' mmm… and I gently had to steer him away to explain why I didn't think that would be a very good idea. (Site 1, C06)*

As described by clinicians, the need to persuade some patients and relatives was the main challenge in the ReSPECT process. To foreclose possibilities for disagreement, some clinicians described structuring ReSPECT conversations to clarify which medical procedures would be undertaken:

> *I think a general structure is this is what's wrong, this is what we will do and this is what we won't do and if they are going to be relevant things like feeding, normal ITU, critical care I think these things need to be discussed. (Site 1, C05)*

As this clinician explained, while they clarified that medical decisions were not open for discussion, they attempted to elicit patients' views during the ReSPECT conversation and integrate these into their decision-making processes:



For peer review only - http://bmjopen.bmj.com/site/about/guidelines.xhtml

*when we are looking at what I think we can do medically we have to take into account what the patient believes [and] how they live their life… (Site 1, C05)*

In line with conversational templates that foreclosed discussion about medical decisions, some clinicians described the ReSPECT conversation as centrally concerned with informing patients and relatives, rather than involving them in medical decision-making. For example, this clinician used the word 'disclose' to describe the function of ReSPECT conversations:

*I still believe it's a medical decision and it's a good practice to inform the patient and their family. So, ultimately, the decision is mine, but I have to disclose my decision to the patient and their family. (Site 2, C15)*

In other interviews, clinicians suggested that, as part of the ReSPECT conversation, clinicians should state explicitly that they are informing patients and relatives about a medical decision, rather than seeking their opinion or approval. One clinician, for example, said that, when conducting a ReSPECT conversation with the relatives of a patient without capacity, one must clarify the relatives' role is to provide contextualising information and ask questions, rather than be actively involved in decision-making:

*I think doctors in particular need to be clear, they're not handing over the decision making to a family member, they are still responsible for the decision but they're ensuring it's made, as far as possible, in line with what the patient would want. (Site 1 C11)*

Another clinician said the ReSPECT form itself, in providing space for patient input, needed to be mediated with care, to avoid conveying that medical decisions required relatives' approval:

*I will normally say that the final decision is a medical decision… 'cause the relatives say 'oh you know I need to check with my brother' when I said that 'I am informing you and just making sure you aware that this is the reason why we are doing it'. (Site 1 C04)*

Along similar lines, a clinician suggested that foregrounding patient views in the ReSPECT conversation was potentially detrimental, as it could place an undue burden on patients or lead to false hope:

*If it's bleeding obvious what can and can't be offered medically then, then you have to be really careful about getting the patient to express about what they want. (…) It has the ironic effect of making them feel more ignored than they would be if, if you just gently explained what is and isn't possible. (Site 1, CO6)*

According to this clinician, asking patients to express their wishes unreservedly was counterproductive. Instead, this clinician argued, doctors should clarify medical possibilities and impossibilities, not place patients in the vulnerable position of having their wishes denied and their hopes deflated.



For peer review only - http://bmjopen.bmj.com/site/about/guidelines.xhtml

Clinicians cited clear and careful communication about the finality of medical decisions as a source of comfort to patients. Describing how they would structure a ReSPECT conversation, one clinician related a hypothetical scenario in which an 82-year-old patient was diagnosed with terminal cancer. In this scenario, they said, they would relate the news to the patient, cite the evidence (as provided by blood tests), and explain what treatments will and will not be offered. Using the second person singular, the clinician described what they would say to this hypothetical patient:

> *"Our aim will be to keep you comfortable, to support you through this. If you have any pain we will, we will control it with strong painkillers. If you have any sickness we will do that. If the time comes and if you stop breathing, or if your heart stops pumping blood… we will not be doing resuscitations, or we will not jump on your chest and perform cardiac compressions because it's not going to work. We will let you go in dignity and respect, and we will support you in that process. We will make sure your family's around you if we can." (Site 2, C16)*

Reflecting on this scenario, they said this approach 'reassured' patients:

> *…if you're very clear to them then they can decide whether they want to be at home, whether they want to be in the hospital. And it just helps them. And if you're quite open to them, they will openly ask you questions and it just makes things easy. (Site 2, C16)*

While most clinicians shared a directive approach to the ReSPECT conversation, particularly in cases where they deemed resuscitation 'futile', it was not the default option for all. One clinician, for example, conceptualized the ReSPECT conversation as 'patient-centred' and as a dialogic process toward a shared decision:

> *So you start off by, by getting the patient to, to give their thoughts on what they would or wouldn't like. And that allows you to, to guide the final decision. Perhaps that's not, so it's not necessarily the patient starting with it. But you do it together. (Site 2, C13)*

Framing the ReSPECT conversation as a dialogue did not preclude medical decision-making. Elsewhere in the interview, this clinician said they initiated ReSPECT conversations with patients they thought should not be for resuscitation. However, this clinician understood the ReSPECT process as complex, often comprised of multiple conversations with clinical and familial actors, building up to a shared decision. This process, they explained, led to deeper understanding and decisions that empowered patients, especially those who decided to forgo future critical care interventions.

**Discussion**

This analysis is the first to explore clinicians' experiences of the ReSPECT process. Focusing on how secondary-care clinicians make decisions about timing and conducting ReSPECT conversations, the findings show that ReSPECT conversations relate to overall treatment plans and are not limited to resuscitation decisions – a major change from the way in which DNACPR decisions have been made to date (Perkins et al., 2016). The analysis finds the management of uncertainty about prognoses



For peer review only - http://bmjopen.bmj.com/site/about/guidelines.xhtml

and patients' emotional reactions is central to clinicians' experiences of ReSPECT conversations. When determining when and with whom to conduct ReSPECT conversations, clinicians rely on their predictions and imaginings of a patient's short-term prognosis, using the ward round as a predictive encounter to manage prognostic uncertainty. When determining which clinician should conduct a ReSPECT conversation and how the conversation should be framed, clinicians seek to maximize rapport and minimize distress, sometimes avoiding or deferring conversations to manage their uncertainty about patients' and relatives' emotional reactions. When deciding whether ReSPECT conversations should inform patients about a clinical decision or involve them in decision-making, clinicians rely on their clarity or uncertainty about patients' trajectories. Throughout, the spatial and temporal constraints of the acute care ward influence clinicians' decisions about which conversations to prioritize and their experiences of rapport with patients.

Many of the findings are consistent with earlier studies on clinicians' experiences of barriers to DNACPR (Holland et al., 2013; Myint et al., 2010) and advance care planning processes (Almack et al., 2012; Sharp et al., 2013). The present analysis, however, is unique in delineating clinicians' approaches to and strategies for overcoming or circumventing these barriers when implementing an ECTP. Moreover, this analysis finds that the central role of uncertainty in ReSPECT conversations both resonates with and diverges from previous research in ways that implicate features particular to ECTPs. Earlier studies have found that negotiating uncertainty is central to medical decision-making and clinical care, particularly when clinicians translate complex population-level evidence to individual prognosis and treatment (Beresford, 1991; Griffiths et al., 2006). Nonetheless, when communicating with patients, clinicians often provide reassurance through discursive modes that convey more certainty than is warranted (Griffiths et al., 2005). The present analysis finds that, when conducting ReSPECT conversations, particularly with patients whose immediate trajectories are unclear, some clinicians present patients and relatives with possible scenarios of future deterioration, to involve them in making advance decisions about clinical care. Yet these expressions of uncertainty about prognosis and treatment, while consistent with the goals of the ECTP informed decision-making process, sometimes clash with patients' and relatives' expectations of reassurance, clinical certainty, and definitive knowledge.

Notably, clinicians explain how they decide when, with whom, and how to conduct a ReSPECT conversation through keywords which include, among others, 'frail', 'futile', and 'co-morbid'. Such keywords serve as shorthand for clinicians' ethical stance on trajectories of treatment. Previous research has found that, on DNACPR forms, clinicians entered keywords such as 'frailty' and 'futility' to justify DNACPR decisions (Cohn et al., 2013). In contrast, on an emergency and advance care planning form (the Universal Form for Treatment Options [UFTO]), such keywords were supplanted by references to specific medical conditions (Fritz et al., 2013). This analysis, however, suggests that clinicians continue to employ these keywords, and the value judgments therein, when making decisions about ReSPECT conversations.

A particular strength of the analysis is the inclusion of clinicians from ten acute care and surgical specialities. This enables the representation of diverse secondary-care environments and clinical attitudes to emergency and advance care planning. Additionally, through its two-stage design, whereby each clinician is first shadowed during a ward round and then interviewed, the analysis allows for an in-depth discussion of ReSPECT conversations in relation to cases observed by the researcher, thus grounding the interview in specific and varied case examples. The analysis is limited



For peer review only - http://bmjopen.bmj.com/site/about/guidelines.xhtml

by its focus on consultants. In both sites, consultants were responsible for signing ReSPECT forms; however, as junior doctors and nurses might take part in ReSPECT conversations, it would have been valuable to include their perspectives and experiences. Finally, as the interviews took place within the first year of ReSPECT implementation in both sites, some findings might reflect experiences related to early implementation.

**Conclusion**

The management of uncertainty about prognoses and patients' emotional reactions is central to secondary-care clinicians' experiences of ReSPECT conversations. When timing ReSPECT conversations, clinicians rely on their predictions of a patient's short-term prognosis; when framing ReSPECT conversations, clinicians seek to minimize distress and maximize rapport; and when involving a patient or a patient's relatives in decision-making discussions, clinicians are guided by their level of certainty about the patient's illness trajectory. Time constraints and busy ward environments interweave with uncertainty to influence clinicians' decisions about which ReSPECT conversations to prioritize. Because the ReSPECT process requires clinicians to hold conversations with patients or their relatives before recording a decision about emergency care and treatments, it may encourage clinicians to articulate decision-making processes and to develop strategies for managing and communicating uncertainty. These findings may be useful to clinicians and organisations implementing ReSPECT, for example, through informing training on how to conduct ReSPECT conversations while facing uncertainty. Further research should explore how clinicians communicate uncertainty, how patients and families experience uncertainty, and how clinicians' experiences of uncertainty relate to the words and values they employ in making decisions about ReSPECT.

For peer review only



For peer review only - http://bmjopen.bmj.com/site/about/guidelines.xhtml

**References**

Almack K, Cox K, Moghaddam N, Pollock K, Seymour J. After you: conversations between patients and healthcare professionals in planning for end of life care. BMC palliative care. 2012 Dec;11(1):15.

Beresford EB. Uncertainty and the shaping of medical decisions. Hastings Center Report. 1991 Jul 8;21(4):6-11.

Braun V, Clarke V. Using thematic analysis in psychology. Qualitative Research in Psychology. 2006;3(2):77-101.

Clements M, Fuld J, Fritz Z. Documentation of resuscitation decision-making: a survey of practice in the United Kingdom. Resuscitation 2014;85:606-11.

Cohn S, Fritz ZBM, Frankau JM, Laroche CM, Fuld JP. Do Not Attempt Cardiopulmonary Resuscitation orders in acute medical settings: a qualitative study. QJM 2013;106:165-77.

Freeman K, Field RA, Perkins GD. Variation in local trust Do Not Attempt Cardiopulmonary Resuscitation (DNACPR) policies: a review of 48 English healthcare trusts. BMJ open 2015;5:e006517.

Fritz Z, Fuld J, Haydock S, Palmer C. Interpretation and intent: a study of the (mis)understanding of DNAR orders in a teaching hospital. Resuscitation 2010;81:1138-41.

Fritz Z, Malyon A, Frankau JM, Parker RA, Cohn S, Laroche CM, Palmer CR, Fuld JP. The Universal Form of Treatment Options (UFTO) as an alternative to Do Not Attempt Cardiopulmonary Resuscitation (DNACPR) orders: a mixed methods evaluation of the effects on clinical practice and patient care. PLoS One. 2013 Sep 4;8(9):e70977.

Fritz Z, Cork N, Dodd A, Maylon A. DNACPR decisions: challenging and changing practice in the wake of the Tracey judgment. Clinical medicine 2014;14:571-6.

Fritz Z, Slowther AM, Perkins GD. Resuscitation policy should focus on the patient, not the decision. BMJ. 2017 Feb 28;356:j813.

General Medical Council. Treatment and care towards the end of life. 2010.

Good BJ, Good MJ, Togan I, Ilbars Z, Güvener A, Gelişen I. In the subjunctive mode: epilepsy narratives in Turkey. Social science & medicine. 1994 Mar 1;38(6):835-42.

Griffiths F, Green E, Tsouroufli M. The nature of medical evidence and its inherent uncertainty for the clinical consultation: qualitative study. BMJ. 2005 Mar 3;330(7490):511.

Griffiths F, Green E, Bendelow G. Health professionals, their medical interventions and uncertainty: A study focusing on women at midlife. Social Science & Medicine. 2006 Mar 1;62(5):1078-90.

McAdam C, Barton A, Bull P, Rai G. An audit of nurses' views on DNR decisions in 1989 and 2003. Br J Nurs 2005;14:1061-2, 4-5.

Mockford C, Fritz Z, George R, Court R, Grove A, Clarke B, Field R, Perkins GD. Do not attempt cardiopulmonary resuscitation (DNACPR) orders: A systematic review of the barriers and facilitators of decision-making and implementation. Resuscitation 2015;88C:99-113.



For peer review only - http://bmjopen.bmj.com/site/about/guidelines.xhtml

Myint PK, Miles S, Halliday DA, Bowker LK. Experiences and views of specialist registrars in geriatric medicine on 'do not attempt resuscitation' decisions: a sea of uncertainty? QJM 2006;99:691-700.

Myint PK, Rivas CA, Bowker LK. In-hospital cardiopulmonary resuscitation: Trainees' worst and most memorable experiences. QJM 2010;103:865-73.

NIHR HS&DR. (2016). Evaluation of the Recommended Summary Plan for Emergency Care and Treatment. https://www.journalslibrary.nihr.ac.uk/programmes/hsdr/151509/#/ [accessed 19 February 2019].

Perkins GD. Do Not Attempt Cardiopulmonary Resusitation (DNACPR) Decisions: Evidence Synthesis. Health Services and Delivery Research 2016; NIHR Journals Library; Vol. 4: No. 11.

Rabkin MT, Gillerman G, Rice NR. Orders not to resuscitate. N Engl J Med 1976;295:364-6.

Resuscitation Council UK. Decisions relating to Cardiopulmonary Resuscitation (3rd edition). 2014. at https://www.resus.org.uk/dnacpr/decisions-relating-to-cpr/.

Sharp T, Moran E, Kuhn I, Barclay S. Do the elderly have a voice? Advance care planning discussions with frail and older individuals: a systematic literature review and narrative synthesis. Br J Gen Pract. 2013 Oct 1;63(615):e657-68.

For peer review only



For peer review only - http://bmjopen.bmj.com/site/about/guidelines.xhtml

**Footnotes**

Patient consent for publication: Not required

Contributors: KE conducted data analysis and drafted the manuscript. AMS, FG and CH designed the study, supervised the data collection, contributed to data analysis and commented on manuscript drafts. GDP and KC contributed to study design, assisted with accessing the field and commented on manuscript drafts. CO collected the data and commented on manuscript drafts. All authors reviewed and approved the final version of the manuscript.

Funding: This article presents independent research funded by the National Institute for Health Research (NIHR) under the Health Services and Delivery Research programme (project number 15/15/09). The views expressed in this publication are those of the authors and not necessarily those of the NIHR or the Department of Health and Social Care. KC is supported by an NIHR post-doctoral research fellowship.

Competing interests: GDP and CH are members of the ReSPECT national working group. AS, FG, CH and GDP received grants from the UK National Institute of Health Research during the study.

Ethics approval: The study received ethics approval from the NRES Committee, West Midlands – Coventry and Warwickshire (REC reference: 17/WM/0134).

Data sharing statement: Although the qualitative data in this study have been pseudonimised, it is possible that with access to raw data individuals might be identifiable. The data are not suitable for sharing beyond what is contained within the manuscript. Further information can be obtained from the corresponding author.

For peer review only

# Reporting checklist for qualitative study.

Based on the SRQR guidelines.

## Instructions to authors

Complete this checklist by entering the page numbers from your manuscript where readers will find each of the items listed below.

Your article may not currently address all the items on the checklist. Please modify your text to include the missing information. If you are certain that an item does not apply, please write "n/a" and provide a short explanation.

Upload your completed checklist as an extra file when you submit to a journal.

In your methods section, say that you used the SRQRreporting guidelines, and cite them as:

O'Brien BC, Harris IB, Beckman TJ, Reed DA, Cook DA. Standards for reporting qualitative research: a synthesis of recommendations. Acad Med. 2014;89(9):1245-1251.

| | | Reporting Item | Page Number |
|---|---|---|---|
| **Title** | | | |
| | #1 | Concise description of the nature and topic of the study identifying the study as qualitative or indicating the approach (e.g. ethnography, grounded theory) or data collection methods (e.g. interview, focus group) is recommended | 1 |

For peer review only

## Abstract

| | | | |
|---|---|---|---|
| | #2 | Summary of the key elements of the study using the abstract format of the intended publication; typically includes background, purpose, methods, results and conclusions | 1 |

## Introduction

| | | | |
|---|---|---|---|
| Problem formulation | #3 | Description and signifcance of the problem / phenomenon studied: review of relevant theory and empirical work; problem statement | 3 |
| Purpose or research question | #4 | Purpose of the study and specific objectives or questions | 3-4 |

## Methods

| | | | |
|---|---|---|---|
| Qualitative approach and research paradigm | #5 | Qualitative approach (e.g. ethnography, grounded theory, case study, phenomenolgy, narrative research) and guiding theory if appropriate; identifying the research paradigm (e.g. postpositivist, constructivist / interpretivist) is also recommended; rationale. The rationale should briefly discuss the justification for choosing that theory, approach, method or technique rather than other options available; the assumptions and limitations implicit in those choices and how those choices influence study conclusions and transferability. As appropriate the rationale for several items might be | 4 |

For peer review only

| | | | |
|---|---|---|---|
| | | discussed together. | |
| Researcher characteristics and reflexivity | #6 | Researchers' characteristics that may influence the research, including personal attributes, qualifications / experience, relationship with participants, assumptions and / or presuppositions; potential or actual interaction between researchers' characteristics and the research questions, approach, methods, results and / or transferability | 4 |
| Context | #7 | Setting / site and salient contextual factors; rationale | 4 |
| Sampling strategy | #8 | How and why research participants, documents, or events were selected; criteria for deciding when no further sampling was necessary (e.g. sampling saturation); rationale | 4 |
| Ethical issues pertaining to human subjects | #9 | Documentation of approval by an appropriate ethics review board and participant consent, or explanation for lack thereof; other confidentiality and data security issues | 18 |
| Data collection methods | #10 | Types of data collected; details of data collection procedures including (as appropriate) start and stop dates of data collection and analysis, iterative process, triangulation of sources / methods, and modification of procedures in response to evolving study findings; rationale | 4 |
| Data collection | #11 | Description of instruments (e.g. interview guides, | 4 |

For peer review only

| | | | |
|---|---|---|---|
| instruments and technologies | | questionnaires) and devices (e.g. audio recorders) used for data collection; if / how the instruments(s) changed over the course of the study | |
| Units of study | #12 | Number and relevant characteristics of participants, documents, or events included in the study; level of participation (could be reported in results) | 4 |
| Data processing | #13 | Methods for processing data prior to and during analysis, including transcription, data entry, data management and security, verification of data integrity, data coding, and anonymisation / deidentification of excerpts | 4-5 |
| Data analysis | #14 | Process by which inferences, themes, etc. were identified and developed, including the researchers involved in data analysis; usually references a specific paradigm or approach; rationale | 4 |
| Techniques to enhance trustworthiness | #15 | Techniques to enhance trustworthiness and credibility of data analysis (e.g. member checking, audit trail, triangulation); rationale | 4 |
| **Results/findings** | | | |
| Syntheses and interpretation | #16 | Main findings (e.g. interpretations, inferences, and themes); might include development of a theory or model, or integration with prior research or theory | 5-13 |
| Links to empirical data | #17 | Evidence (e.g. quotes, field notes, text excerpts, photographs) to substantiate analytic findings | 5-13 |

For peer review only

## Discussion

| | | | |
|---|---|---|---|
| Intergration with prior work, implications, transferability and contribution(s) to the field | #18 | Short summary of main findings; explanation of how findings and conclusions connect to, support, elaborate on, or challenge conclusions of earlier scholarship; discussion of scope of application / generalizability; identification of unique contributions(s) to scholarship in a discipline or field | 13-14 |
| Limitations | #19 | Trustworthiness and limitations of findings | 14-15 |

## Other

| | | | |
|---|---|---|---|
| Conflicts of interest | #20 | Potential sources of influence of perceived influence on study conduct and conclusions; how these were managed | 18 |
| Funding | #21 | Sources of funding and other support; role of funders in data collection, interpretation and reporting | 18 |

The SRQR checklist is distributed with permission of Wolters Kluwer © 2014 by the Association of American Medical Colleges. This checklist was completed on 13. May 2019 using https://www.goodreports.org/, a tool made by the EQUATOR Network in collaboration with Penelope.ai

For peer review only

BMJ Open

# Secondary Care Consultant Clinicians' Experiences of Conducting Emergency Care and Treatment Planning Conversations in England: An Interview-Based Analysis



| | |
|---|---|
| Journal: | *BMJ Open* |
| Manuscript ID | bmjopen-2019-031633.R1 |
| Article Type: | Original research |
| Date Submitted by the Author: | 09-Sep-2019 |





| | |
|---|---|
| Complete List of Authors: | Eli, Karin; University of Warwick, Division of Health Sciences, Warwick Medical School<br>Ochieng, Cynthia; Cardiff University, School of Medicine<br>Hawkes, Claire; University of Warwick Warwick Medical School, Clinical Trials Unit<br>Perkins, Gavin; University of Warwick, Clinical Trials Unit<br>Couper, Keith; University of Warwick, Warwick Medical School<br>Griffiths, Frances; University of Warwick, Warwick Medical School<br>Slowther, Anne-Marie; University of Warwick, Warwick Medical School |





| | |
|---|---|
| <b>Primary Subject Heading</b>: | Health services research |
| Secondary Subject Heading: | Patient-centred medicine, Qualitative research |
| Keywords: | Emergency care treatment planning, Advance care planning, Clinicians' experiences, Do not attempt cardiopulmonary resuscitation (DNACPR), QUALITATIVE RESEARCH, Decision making |

SCHOLARONE™ Manuscripts

# Secondary Care Consultant Clinicians' Experiences of Conducting Emergency Care and Treatment Planning Conversations in England: An Interview-Based Analysis

Karin Eli[1], Cynthia Ochieng[2], Claire Hawkes[1], Gavin D. Perkins[1, 3], Keith Couper[1, 3], Frances Griffiths[1]*, Anne-Marie Slowther[1]*

[1] Warwick Medical School, University of Warwick
[2] School of Medicine, Cardiff University
[3] Critical Care Unit, University Hospitals Birmingham NHS Foundation Trust, Birmingham

*These authors share senior authorship

Corresponding author: Karin Eli (Karin.Eli@warwick.ac.uk)

For peer review only

**Abstract**

Objective: To examine secondary-care clinicians' experiences of conducting conversations about treatment escalation with patients and their relatives, using the Recommended Summary Plan for Emergency Care and Treatment (ReSPECT) process.

Design: Semi-structured interviews following ward round observations.

Setting: Two National Health Service hospitals in England.

Participants: Fifteen medical and surgical consultants from 10 specialties, observed in 14 wards.

Analysis: Interview transcripts were analysed using thematic analysis.

Results: Three themes were developed: (1) Determining when and with whom to conduct a ReSPECT conversation; (2) Framing the ReSPECT conversation to manage emotions and relationships; and (3) Reaching ReSPECT decisions. The results showed that when timing ReSPECT conversations, clinicians rely on their predictions of a patient's short-term prognosis; when framing ReSPECT conversations, clinicians seek to minimize distress and maximize rapport; and when involving a patient or a patient's relatives in decision-making discussions, clinicians are guided by their level of certainty about the patient's illness trajectory.

Conclusions: The management of uncertainty about prognoses and about patients' emotional reactions is central to secondary-care clinicians' experiences of timing and conducting ReSPECT conversations.

**Keywords**

Emergency care treatment planning; advance care planning; clinicians' experiences; critical care; do not attempt cardiopulmonary resuscitation (DNACPR); decision making; qualitative research; recommended summary plan for emergency care and treatment (ReSPECT); uncertainty.

**Article summary: strengths and limitations of this study**

- Data were collected from clinicians from ten medical specialities, thereby representing diverse secondary-care environments and clinical attitudes to emergency care and treatment planning.



For peer review only - http://bmjopen.bmj.com/site/about/guidelines.xhtml

- Each clinician was first shadowed during a ward round and then interviewed, thus grounding the interviews in specific and varied case examples.
- The findings reported in this paper are limited by the study's focus on consultants' interviews; as other members of multidisciplinary teams also participate in ReSPECT conversations, including their perspectives and experiences would have been valuable.
- The interviews took place within the first year of ReSPECT implementation in the two study sites, such that some findings may reflect experiences related to early implementation.

For peer review only



For peer review only - http://bmjopen.bmj.com/site/about/guidelines.xhtml

**Introduction**

Most DNACPR decisions are made when clinicians determine that the burdens of cardiopulmonary resuscitation (CPR) for the patient outweigh potential benefits. However, DNACPR decisions can also be made when clinicians predict that CPR would not succeed, or when a patient requests not to be resuscitated.[1 2] While DNACPR guidelines are clearly articulated, several studies and reviews have found that, in practice, DNACPR processes are fraught with ambiguity. Clinicians have varying, sometimes-divergent understandings of DNACPR decision-making processes, leading to inconsistencies in how decisions are made, implemented, and recorded.[3-6] These inconsistencies may lead to lower quality of care; indeed, some clinicians misinterpret DNACPR decisions as limiting other aspects of treatment, while others administer CPR inappropriately, failing to follow patients' wishes for the withholding of resuscitation.[3 7-10] Notably, clinicians often communicate poorly about DNACPR with patients and their relatives, and some are reluctant to discuss resuscitation, thereby excluding patients from the decision-making process.[4 6 7 11-13]

This paper is part of a larger study, funded by the National Institute for Health Research (NIHR), which evaluates the Recommended Summary Plan for Emergency Care and Treatment (ReSPECT).[14] Launched in 2017 across NHS Trusts, ReSPECT is an emergency care treatment plan (ECTP) developed in response to the gaps observed in the DNACPR process. ReSPECT builds on research conducted in the US, the UK, and Canada, which found that programmes that integrate DNACPR with discussions about wider goals of treatment increase clarity about trajectories of care and reduce harm to patients.[15] As an ECTP which records clinical recommendations that take into account patients' values and preferences, ReSPECT places resuscitation within a wider context of treatments that should or should not be considered in an emergency situation.

The authors of ReSPECT emphasise that it is a process designed to guide clinicians in discussing with patients what might be optimal treatment choices for them with the ReSPECT form acting as a prompt and summary record of the discussion and its outcomes.[16] The form and its associated guidance documents were developed in 2016 by the ReSPECT working group. Chaired by the Resuscitation Council (UK) and Royal College of Nursing, the ReSPECT working group had representation from patients, professional organisations (Royal Colleges, British Medical Association) regulatory bodies (General Medical Council, Nursing Midwifery Council), the Care Quality Commission, NHS organisations (Acute, Community and Ambulance Trusts) and patient and public members. The completed ReSPECT form is held by the patient, allowing them to communicate the treatment plans when they move from one healthcare setting to another.

In the present paper, we report findings from interviews with secondary-care consultant clinicians in two NHS organisations that had recently implemented ReSPECT, exploring why, when, and with whom they choose to have ReSPECT conversations. Our aim is to inform future development of the process and the current implementation across the NHS and to provide focus to further qualitative research on how ReSPECT becomes integrated into health professionals' practice.



For peer review only - http://bmjopen.bmj.com/site/about/guidelines.xhtml

**Methods**

Fifteen consultants (six female, nine male) from two acute NHS teaching hospitals in England were interviewed from August to December 2017 as part of a wider ongoing study, aimed at evaluating the implementation of the ReSPECT process. The 15 consultants represented 14 wards and 10 medical specialities. Interviews took place in site one 7-10 months after ReSPECT had been implemented and in Site two, 11-12 months after implementation. We observed no differences related to ReSPECT implementation timelines between the two sites.

Potential participants were identified by the local principal investigator at each of the participating hospitals through purposive sampling designed to represent a range of views about the ReSPECT process, as well as a diversity of clinical areas that could be replicated across NHS trusts (three medical specialities, a surgical speciality, and orthopaedics). The local PIs or research nurses asked for volunteer participants from these specialities and the study's research fellow scheduled ward round observations directly with the participating consultants, to ensure that observations did not place an undue burden on their clinical practice. All participating consultants provided written informed consent prior to taking part in the study.

The research fellow, a public health researcher, shadowed each consultant during a ward round, to observe when and how consultants engaged in ReSPECT conversations with their patients. Shadowing is a structured observation technique[17] which has been identified as appropriate for qualitative research on clinicians' experiences and practices.[18 19] To ensure that patients, relatives, and staff were aware that observations were taking place, study posters were displayed in the selected wards, and the research fellow wore a scrubs uniform top with the word 'researcher' printed clearly on both the front and the back. During each shadowing period the participating consultant introduced the researcher to each patient (and family if appropriate) and informed them that they could request that the researcher leave if they wished. A brief information leaflet was left with the patient. The researcher interviewed each consultant following the observation, typically within 24 -48 hours. The interviews were semi-structured and were designed to explore each consultant's decision-making about holding a ReSPECT conversation in three observed cases, as well as the consultant's wider experiences with ReSPECT. If the researcher observed three ReSPECT conversations (which was the maximum she had observed in any of her observation sessions), she selected these three cases for discussion during the interviews. If she observed fewer than three ReSPECT conversations, she selected one or more cases where she thought a ReSPECT conversation might have been appropriate, to explore with the clinician why they chose not to hold a ReSPECT conversation in those cases. The interview topic areas were developed based on the study's research questions and the literature, and the observation and interview approach was checked with members of the study team with relevant clinical experience. The interviews lasted from 15 to 53 minutes, with a median time of 37 minutes, and were digitally recorded and transcribed.

Interview transcripts were analysed by the study's senior research fellow (SRF), a medical anthropologist, using thematic analysis.[20] First, the SRF read the interview transcripts to identify initial codes. The transcripts were then coded closely, with most codes developed at the level of sentences or sentence clauses. The SRF reviewed the coded interviews, and grouped the codes to develop themes. The themes were continuously revised throughout the process of reviewing the coded interviews, leading to 16 emerging themes, which were grouped into overarching themes. To



For peer review only - http://bmjopen.bmj.com/site/about/guidelines.xhtml

ensure intercoder reliability, four of the 15 interviews were analysed independently by another SRF, a health services researcher. The two SRFs discussed the codes, identified differences and potential disagreements, and discussed these until they reached consensus. In total, five initial overarching themes were developed: three focused on the ReSPECT conversation, one focused on consultants' value judgments, and one focused on the ReSPECT form. After they achieved consensus, the SRFs discussed the five overarching themes with two of the senior co-authors, doctors with research expertise in medical ethics and medical sociology. Together, they decided to focus the analysis on the three overarching themes concerned with the ReSPECT conversation, as these themes most closely responded to the study's aim of exploring why, when, and with whom consultants choose to have ReSPECT conversations. Finally, the first SRF reviewed all interview transcripts to ensure the three themes represented the data accurately. Throughout the analytic process, coding was conducted using word processing software. To maintain participant confidentiality, the gender-neutral pronouns they/them are used to refer to all consultants.

Patient and public involvement (PPI)

The study has been supported throughout by a PPI group, which informed the study design and the development of models of recruitment and consent. Additionally, PPI group members provided feedback on a draft of the manuscript. They agreed with the manuscript's findings and offered suggestions for areas to investigate further in our future research, in light of their own experiences as patients or carers.

## Results

*Theme 1: Determining when and with whom to conduct a ReSPECT conversation: Uncertainty management and catalysts for discussion*

Given time constraints, consultants had to determine which patients were most in need of a ReSPECT conversation, and when this conversation should be conducted. Making these determinations was fraught with uncertainty. To manage this uncertainty, consultants relied on their predictions and imaginings of patients' immediate futures, using the ward round to piece together prognostic puzzles. For example, explaining why he did not initiate a ReSPECT conversation with a patient in her 90s, this consultant said:

> *she's otherwise recovering well (…) I thought the chances of her, as it were, needing any of the interventions you might discuss on a ReSPECT form were small. (Site 1, C10)*

The key to initiating a ReSPECT conversation, this consultant later explained, was predicting a trajectory of deterioration:

> *It's the deteriorating patients, patients with end-of-life conditions for whatever reason, be it cancer or organ failure and any patient where they might suddenly deteriorate. (Site 1, C10)*

Another consultant said they used the ward round to predict whether a patient was likely to experience a 'catastrophe'; such a prediction, they said, would warrant initiating a ReSPECT conversation:



For peer review only - http://bmjopen.bmj.com/site/about/guidelines.xhtml

*Particularly where you're seeing acutely ill patients and you're seeing them for the first time as, as an acute physician, I think the prompt is how likely you think it is that this patient may have a catastrophe, may have a cardiac arrest, may have a sudden severe deterioration. (Site 1, C11)*

This consultant, like numerous others in the sample, linked the focus on predicted deterioration to the time constraints of the ward round. As another consultant explained:

*I think in the context of a post-take ward round where I am time limited I prioritise those patients for whom these conversations are most likely to be required for this admission. So it may well be that ReSPECT conversations were appropriate for more of the patients that I saw today in terms of potentially being last year of life. But they were not decisions that were required today. (Site 2, C17)*

While time constraints were frequently cited, they were not the only factor underlying consultants' focus on predicted deterioration. Explaining why they were less likely to initiate a ReSPECT conversation with some patients, this consultant positioned their decision-making as culturally embedded:

*I think for the time being the culture is still the ReSPECT form is mainly for when people deteriorate. I think sometimes asking people a hypothetical question when they're really quite well, it's difficult to frame it. (Site 1, C09)*

This consultant linked their focus on deterioration as the primary prompt for a ReSPECT conversation to the difficulty of asking patients to imagine a hypothetical catastrophic scenario. Imagining difficult scenarios, however, was central to ReSPECT conversations. Since initiating ReSPECT conversations depended on clinicians' predictions of patients' short-term prognoses, ReSPECT conversations engaged patients with clinicians' predictive thinking. This process was often challenging, as patients did not necessarily share in the logics and concepts of time posed by their clinicians:

*[P]eople find, "What if?" challenging. So if I say, "What if you're going to deteriorate? We need to make a decision what we would do about ITU." A lot of patients and relatives will hear about us saying, "You're deteriorating, you're going to need ITU." They don't hear the "What if?" (Site 2, C17)*

Like others, this consultant explained that while they framed possible future scenarios in subjunctive – that is, potential or conditional – terms, patients and relatives tended to understand these in definitive future terms. Another consultant explained that, rather than joining a dialogue on potential scenarios, some patients and relatives expressed distress over what they understood as a terminal prognosis:

*…even though I've said "I am going to let you go home now, have you thought about what you would like in the future?", and then they say "why am I going to die? You're telling me I am going to die aren't you!" (Site 1, C01)*



For peer review only - http://bmjopen.bmj.com/site/about/guidelines.xhtml

In addition to predicted deterioration, consultants timed ReSPECT conversations according to calculations of risk related to a wider network of actors. The timing of ReSPECT conversations therefore implicated not only patients, but also other clinicians. For example, some consultants said they were reluctant to hold ReSPECT conversations with pre-operative patients, as these discussions could bias surgeons or demoralise patients:

> *[The patient] was going to undergo an operation and I feel very uncomfortable discussing resuscitation just before the operation (…) if he does not want to be resuscitated, it influences the surgeon to some extent. (Site 2, C14)*

> *…we don't often talk about ReSPECT form because it's, you know, when you deteriorate. And in some ways with elective surgery they're very much focused on consenting for surgery and talking about rehab after surgery rather than deterioration from surgery. (Site 1, C08)*

Elsewhere in the interview, this surgeon said the best time to initiate a ReSPECT conversation was immediately after surgery – a particularly opportune time because, while risk of complications was higher after surgery, patients' distress was likely to be lower, and relatives were likely to be present:

> *…often, often there's a family around at that point because it's usually an emergency admission and the family come in a day or two later. So you can involve the parties that you need to at that point in time. It's a relatively rare event for a patient to die on the operating table, if they're going to, if they're going to succumb it's usually over the following few days. (Site 1, C08)*

The presence of relatives was central to the timing of many ReSPECT conversations, not least because conducting the ReSPECT process with patients who lack capacity requires the participation of an individual close to the patient. In this example, a consultant discussed a case where a visit from a patient's relatives prompted a ReSPECT conversation:

> *[H]is family were there so I took the opportunity while they were all there to express not only that he was perhaps more unwell than they had recognised, and that he was getting better with treatment, and to explore what their feelings were about escalation of care, particularly whether intensive care would be appropriate for him. (Site 2, C17)*

This consultant considered the presence of relatives crucial in timing the ReSPECT conversation due to uncertainty about escalating the patient's care. Other consultants, however, spoke of the presence of relatives as important for finalizing and communicating a medical decision, rather than deliberating about a trajectory of care.

*Theme 2: Conducting the ReSPECT conversation: Managing emotions and relationships*

Most ReSPECT conversations implicated a triad of patient, clinician(s), and relative(s). For patients who lacked capacity, this triad was essential to the ReSPECT process, with relatives or other advocates called upon to speak on the patient's behalf. However, while patients with capacity could speak privately with their clinicians, they often involved their relatives, framing the ReSPECT



For peer review only - http://bmjopen.bmj.com/site/about/guidelines.xhtml

conversation and their own decision-making as familial. In these cases, consultants clarified they included relatives in the conversation, but did not involve them in decision-making:

> *…you've had a discussion, the patient says, "Look, I don't want anything done, doctor," I think it's still very valuable to the next of kin to know that. (…) but we're not asking the family to participate in the discussion if the patient has already made their wishes clear in a reliable way. (Site 1, C11)*

Although consultants tended to describe relatives' involvement as valuable, they also described it as potentially problematic, characterising family members as either compliant and 'sensible', or as non-compliant and 'difficult'. According to some consultants, relatives sometimes challenged clinical decisions – specifically, decisions against resuscitation – because they misunderstood what 'not for resuscitation' meant for the patient's future care:

> *Sometimes you have relatives who are very emotional, sometimes they think when you say 'not for resuscitation' means you're going to stop all treatment. (Site 2, C16)*

In other cases, consultants said relatives misunderstood their role in the ReSPECT conversation as that of 'decision-maker', worrying about how a 'not for resuscitation' decision might reflect on them:

> *Often what happens is the relatives feel that you're asking them to make the decision… and again because they've been misled by the media, they feel that if they say, yes, make them not for resuscitation, that they might be seen as a money grabbing. (Site 2, C12)*

Disagreement between clinicians and patients' relatives could carry consequences for patient care, particularly if relatives who held legal power of attorney (LPA) attempted to overturn a clinical decision. In those cases, consultants advocated for their clinical decision, taking the role of acting on the patient's behalf:

> *I try to explain to them that by keeping them alive, you are, you are, you are prolonging their agony. (…) I try to avoid confrontation with them (…) But sometimes we have to, when I can see clearly that there is going to be harm, then I have to, even if they have the LPA. (Site 2, C14)*

Another relational aspect of the ReSPECT conversation was the consideration of other, sometimes absent, clinicians. Several consultants spoke about the importance of identifying the 'right' clinician to conduct a ReSPECT conversation – often, the consultant or the GP regularly charged with the patient's care. In post-take ward rounds, some consultants avoided conducting ReSPECT conversations with patients who were usually seen by their colleagues. In this example, a consultant explained why they chose not to complete a ReSPECT form with a patient who had a localised infection:

> *I could've completed a ReSPECT form but I didn't because I, effectively I'm not looking at her [as her] responsible consultant. (Site 2, C12)*



For peer review only - http://bmjopen.bmj.com/site/about/guidelines.xhtml

Later in the interview, this consultant explained that the patient's condition did not warrant an urgent ReSPECT conversation. Given the lack of urgency, they deferred to the patient's 'usual physicians, who obviously know her prognosis'. It would be inappropriate, this consultant argued, to conduct a ReSPECT conversation with a patient whose consultants evidently did not deem it necessary.

Consultants deferred ReSPECT conversations until the 'right' clinicians could conduct them not only because patients' usual consultants were more knowledgeable about these patients' medical histories, but also because these usual consultants had established rapport with the patients. In this example, a consultant explained why they chose to conduct a ReSPECT conversation but leave the final decision for a future discussion between the patient and her usual consultant. The patient, this consultant explained, was not 'receptive' to an earlier ReSPECT conversation with her usual consultant. As such, they viewed their role as providing a second opinion to support the consultant's, rather than as finalizing a ReSPECT decision.

> *…I didn't feel as if I was going to be welcomed to take that further with her myself. So I thought it was better than to say, to see her back to her normal consultant then the next time. (Site 2, C13)*

For similar reasons, other consultants suggested that ReSPECT conversations were best conducted in primary care settings, led by patients' GPs rather than by clinicians they first met during an acute care admission. In response to the researcher's question, 'So you think this is something that should be discussed in the community?', this consultant said:

> *Definitely because I think it makes… patients feel less vulnerable… when they are in hospital they feel vulnerable plus they don't know us (…) they might have known the GP or have some sort of on-going or community matron or something that's a bit more of a long term relationship. (Site 1, C04)*

The importance consultants placed on rapport was closely connected to their concerns over trustworthiness. Worries about being perceived as untrustworthy led some consultants to avoid or delay ReSPECT conversations with some patients. As described by consultants, ReSPECT conversations, if not framed carefully, could undermine the process of building trust with patients.

> *One of my worries is that patients, if you're not careful with your language, a patient might interpret a discussion about what to do in the event of deterioration, escalation, CPR, etcetera, as you giving up on them, as you not being prepared to do everything that you can to get them over their illness. (Site 1, C11)*

The timing of ReSPECT conversations could also affect trust building. This consultant, for example, suggested that initiating a ReSPECT conversation too early would shake the patient's trust:

> *… you want to make sure you still have the rapport with the patient, that they see you as somebody that's there to help them (…) and if you feel that the patient is not quite ready to*



For peer review only - http://bmjopen.bmj.com/site/about/guidelines.xhtml

*talk about it or they don't want to then if you kind of push it they'll see you negatively. (Site 1, C04)*

Another consultant, a surgeon, described a case where they conducted a ReSPECT conversation before operating on a seriously ill patient. While the ReSPECT conversation was carefully timed from a medical perspective, it forced the patient to confront difficult scenarios that destabilized her trust in the surgeon:

*So she doesn't want to talk about whether or not she's going to die on the operating table, or whether or not she's going to get her post-op chest infection or a lung embolus or whatever else could happen. But that process makes us talk about it at that point in time. (…) as soon as you mention that sentence about what would you like to do and if things were taking a turn for the worse (…) she's switched, she's completely switched off. (Site 1, C08)*

Notably, consultants were concerned about being perceived as trustworthy because they identified the ReSPECT conversation as a catalyst for potential distress for patients and relatives. To manage the difficult emotions that often arose during ReSPECT conversations, consultants used various techniques: from avoiding the conversation if the patient was expected to react aggressively or become overwhelmed, to initiating a series of conversations to ease patients and relatives into their future trajectory. For example, one consultant deferred ReSPECT conversations with patients recently diagnosed with terminal cancer to avoid overwhelming them:

*I've generally just told them they've got incurable cancer and it, to go on straight from that to a ReSPECT conversation is too much. But I will say that it exists and that it may be something they want to consider and then ask somebody else to follow it up. (Site 2, C17)*

Alongside concerns over patients' emotional wellbeing, several consultants said that previous experiences with patients or relatives who became upset made them cautious about initiating and framing ReSPECT conversations. One consultant, who explained that 'we worry about the angry and anxious one[s]' (Site 2, C17), described beginning each ReSPECT conversation by framing it as common and routine, to pre-empt patients' upset reactions. Another consultant, who described ReSPECT conversations as 'emotionally very draining', conducted repeated ReSPECT conversations to manage relatives' distress:

*So if you can get some background knowledge, and if they are so in shock that they can't take anything in then it's okay to come back another time. (…) I would prefer to sit away in a, in a room together with a nurse accompanying me, so that you've got a bit of time to yourself and make sure that they know you've got time to listen to them and questions and things. (Site 1, C09).*

The availability of sufficient time and adequate space influenced consultants' capacity to conduct ReSPECT conversations. Many conversations, of necessity, took place during ward rounds, and the crowdedness, urgent pace, and lack of privacy in acute wards limited clinicians' ability to conduct the in-depth ReSPECT conversations they envisioned as appropriate. This consultant, for example,



For peer review only - http://bmjopen.bmj.com/site/about/guidelines.xhtml

argued that ReSPECT conversations necessitated the quiet environment of the patient's home or GP surgery:

> *…this is quite a serious and significant discussion that should not take place in a very busy, busy place. It should either happen when the patient is comfortable in their own home, or, or they have gone to see their, gone to see their GP… (Site 2, C14)*

Hectic ward environments, in this consultant's experience, implicated an urgency and sensory onslaught that, together with patients' acute conditions, led to compromised conversations. The lack of sufficient time to conduct ReSPECT conversations in acute care wards was a pervasive concern across the sample:

> *…it takes time and it sort of stirs up emotions both in you and in the patient (…) so it can be very difficult, mmm, not least because you want to do it well and yet you know we were on a ward round which isn't an ideal kind of, ideally you'd come back and spend 20 minutes with each of them wouldn't you and their families and talk to them at some length. (Site 1, C06)*

Notably, this consultant suggested that lack of time was not simply a logistical issue, but a factor that reduced the ability to conduct careful ReSPECT conversations and manage the emotions that arose during ReSPECT conversations.

<u>*Theme 3: Reaching ReSPECT decisions: Involving versus informing*</u>

The extent to which ReSPECT conversations engaged with patients' wishes depended on consultants' clarity or uncertainty about patients' trajectories. When consultants had clear predictions for patients' short-term prognoses, they tended to lead ReSPECT conversations, taking an informative and persuasive stance. For example, when asked by the researcher, 'Are there times when you find yourself pushing the discussion in a particular way?', this consultant responded:

> *Yes, I think if you genuinely feel that it would be completely futile and that you would only be prolonging an unpleasant death then yes, you do, you do tend to push the discussion in one way or another. (Site 1, C10)*

Consultants often used words such as 'futile', 'frail', or 'co-morbid' when describing cases in which they took a persuasive stance. Futility, as consultants framed it, foreclosed discussion of patient preferences. The conversation focused on patient preferences only when consultants were uncertain about a patient's trajectory:

> *I think that the times where it's very important to discuss with a patient whether they would be appropriate for resuscitation is if it's a patient that maybe is potentially a candidate for intensive care, Level 3 care, that isn't so frail and co-morbid that we feel it would be utterly futile. (Site 2, C12)*

Because they approached ReSPECT conversations according to perceptions of prognostic clarity and uncertainty, many consultants described the ReSPECT conversations in which they typically engaged

For peer review only



For peer review only - http://bmjopen.bmj.com/site/about/guidelines.xhtml

– conversations with patients at imminent risk – as processes of navigation and persuasion. For example, one consultant described handling a patient's son's concerns by 'steer[ing]' the conversation:

> *I went in with quite clear views of what had to be done and as you say the patient's son started to suggest that 'actually he would want to be resuscitated wouldn't you Dad' mmm… and I gently had to steer him away to explain why I didn't think that would be a very good idea. (Site 1, C06)*

As described by consultants, the need to persuade some patients and relatives was the main challenge in the ReSPECT process. To foreclose possibilities for disagreement, some consultants described structuring ReSPECT conversations to clarify which medical procedures would be undertaken:

> *I think a general structure is this is what's wrong, this is what we will do and this is what we won't do and if they are going to be relevant things like feeding, normal ITU, critical care I think these things need to be discussed. (Site 1, C05)*

As this consultant explained, while they clarified that medical decisions were not open for discussion, they attempted to elicit patients' views during the ReSPECT conversation and integrate these into their decision-making processes:

> *when we are looking at what I think we can do medically we have to take into account what the patient believes [and] how they live their life… (Site 1, C05)*

In line with structuring conversations to foreclose debate about medical decisions, some consultants described the ReSPECT conversation as centrally concerned with informing patients and relatives, rather than involving them in medical decision-making. For example, this consultant used the word 'disclose' to describe the function of ReSPECT conversations:

> *I still believe it's a medical decision and it's a good practice to inform the patient and their family. So, ultimately, the decision is mine, but I have to disclose my decision to the patient and their family. (Site 2, C15)*

In other interviews, consultants suggested that, as part of the ReSPECT conversation, clinicians should state explicitly that they are informing patients and relatives about a medical decision, rather than seeking their opinion or approval. One consultant, for example, said that, when conducting a ReSPECT conversation with the relatives of a patient without capacity, one must clarify the relatives' role is to provide contextualising information and ask questions, rather than be actively involved in decision-making:

> *I think doctors in particular need to be clear, they're not handing over the decision making to a family member, they are still responsible for the decision but they're ensuring it's made, as far as possible, in line with what the patient would want. (Site 1 C11)*



For peer review only - http://bmjopen.bmj.com/site/about/guidelines.xhtml

Another consultant said the ReSPECT form itself, in providing space for patient input, needed to be mediated with care, to avoid conveying that medical decisions required relatives' approval:

> *I will normally say that the final decision is a medical decision… 'cause the relatives say 'oh you know I need to check with my brother' when I said that 'I am informing you and just making sure you aware that this is the reason why we are doing it'. (Site 1 C04)*

Along similar lines, a consultant suggested that foregrounding patient views in the ReSPECT conversation was potentially detrimental, as it could place an undue burden on patients or lead to false hope:

> *If it's bleeding obvious what can and can't be offered medically then, then you have to be really careful about getting the patient to express about what they want. (…) It has the ironic effect of making them feel more ignored than they would be if, if you just gently explained what is and isn't possible. (Site 1, CO6)*

According to this consultant, asking patients to express their wishes unreservedly was counterproductive. Instead, this consultant argued, doctors should clarify medical possibilities and impossibilities, not place patients in the vulnerable position of having their wishes denied and their hopes deflated.

Consultants cited clear and careful communication about the finality of medical decisions as a source of comfort to patients. Describing how they would structure a ReSPECT conversation, one consultant related a hypothetical scenario in which an 82-year-old patient was diagnosed with terminal cancer. In this scenario, they said, they would relate the news to the patient, cite the evidence (as provided by blood tests), and explain what treatments will and will not be offered. Using the second person singular, the consultant described what they would say to this hypothetical patient:

> *"Our aim will be to keep you comfortable, to support you through this. If you have any pain we will, we will control it with strong painkillers. If you have any sickness we will do that. If the time comes and if you stop breathing, or if your heart stops pumping blood… we will not be doing resuscitations, or we will not jump on your chest and perform cardiac compressions because it's not going to work. We will let you go in dignity and respect, and we will support you in that process. We will make sure your family's around you if we can." (Site 2, C16)*

Reflecting on this scenario, they said this approach 'reassured' patients:

> *…if you're very clear to them then they can decide whether they want to be at home, whether they want to be in the hospital. And it just helps them. And if you're quite open to them, they will openly ask you questions and it just makes things easy. (Site 2, C16)*

While most consultants shared a directive approach to the ReSPECT conversation, particularly in cases where they deemed resuscitation 'futile', it was not the default option for all. One consultant, for example, conceptualized the ReSPECT conversation as 'patient-centred' and as a dialogic process toward a shared decision:



For peer review only - http://bmjopen.bmj.com/site/about/guidelines.xhtml

*So you start off by, by getting the patient to, to give their thoughts on what they would or wouldn't like. And that allows you to, to guide the final decision. Perhaps that's not, so it's not necessarily the patient starting with it. But you do it together. (Site 2, C13)*

Framing the ReSPECT conversation as a dialogue did not preclude medical decision-making. Elsewhere in the interview, this consultant said they initiated ReSPECT conversations with patients they thought should not be for resuscitation. However, this consultant understood the ReSPECT process as complex, often comprised of multiple conversations with clinical and familial actors, building up to a shared decision. This process, they explained, led to deeper understanding and decisions that empowered patients, especially those who decided to forgo future critical care interventions.

**Discussion**

Our analysis found that the management of uncertainty about prognoses and patients' and relatives' emotional reactions is central to consultants' experiences of ReSPECT conversations. When determining when and with whom to conduct ReSPECT conversations, consultants rely on their predictions of a patient's short-term prognosis, prioritising patients for whom they are certain treatment escalation would not be medically indicated. When patients lack capacity, consultants also time conversations to coincide with the presence of patients' relatives, underscoring the importance of involving next of kin in these conversations, as specified in English law.[21] When determining which clinician should conduct a ReSPECT conversation and how the conversation should be framed, consultants seek to maximize rapport and minimize distress, sometimes avoiding or deferring conversations to manage uncertainty about patients' and relatives' emotional reactions. When deciding whether ReSPECT conversations should inform patients about a clinical decision or involve them in decision-making, consultants rely on their clarity or uncertainty about patients' trajectories. Thus, consultants' decisions about with whom to have RESPECT conversations, when to have these conversations, and whether to frame these conversations as explaining medical decisions or as eliciting patients' preferences are driven by consultants' degrees of uncertainty about prognoses, reactions, and outcomes. Throughout, the time-pressured and busy environments of acute care wards influence consultants' decisions about which conversations to prioritise and their experiences of rapport with patients.

Many of the findings are consistent with earlier studies on clinicians' experiences of barriers to DNACPR[10 22] and ACP processes.[23 24] Notably, the findings resonate with a recent systematic review of qualitative studies on the implementation of ACPs, which found that clinicians' uncertainty about prognoses, uncertainty about patients' and relatives' reactions to ACP, and structural constraints related to the clinical environment all constituted barriers to ACP processes.[25] The finding of the central role of uncertainty in ReSPECT conversations both resonates with and diverges from previous research in ways that implicate features particular to ECTPs. Earlier studies have found that negotiating uncertainty is central to medical decision-making and clinical care, particularly when clinicians translate complex population-level evidence to individual prognosis and treatment.[26 27] Nonetheless, when communicating with patients, clinicians often provide reassurance through discursive modes that convey more certainty than is warranted.[28] The present analysis finds that,



For peer review only - http://bmjopen.bmj.com/site/about/guidelines.xhtml

when conducting ReSPECT conversations, particularly with patients whose immediate trajectories are unclear, some consultants present patients and relatives with possible scenarios of future deterioration, to involve them in the decision-making process. Yet these expressions of uncertainty about prognosis and treatment, while consistent with the goals of the ECTP sometimes clash with patients' and relatives' expectations of reassurance, clinical certainty, and definitive knowledge. Previous research has suggested that clinicians can frame expressions of uncertainty productively, as an opening to shared decision-making discussions with patients.[29] Based on the present study's findings, training clinicians in how to frame uncertainty as a conversational prompt may be of particular importance in the implementation of ReSPECT.

Notably, consultants explained how they decide when, with whom, and how to conduct a ReSPECT conversation through keywords which include, among others, 'frail', 'futile', and 'co-morbid'. Such keywords may serve as shorthand for clinicians' ethical stance on trajectories of treatment, although 'frail' and 'co-morbid' may also express clinical assessment. The use of such keywords without reference to clinical assessments may therefore be potentially problematic; 'futility', in particular, has been subject to debate within the medical ethics literature, with some authors arguing that the use of this term, for which no consensus definition exists, can muddle decision-making and hinder patient autonomy.[30] Previous research has found that, on DNACPR forms, clinicians entered keywords such as 'frailty' and 'futility' to justify DNACPR decisions.[9] This analysis suggests that clinicians continue to employ these keywords. How doctors are using these keywords in the context of ReSPECT conversations warrants further exploration.

One aim of the ReSPECT process is to move discussions of future emergency treatment from a focus on CPR to broader considerations of potential treatments. Our analysis shows that some consultants are broadening these discussions. However, in the early adoption phase of ReSPECT, is seems that many conversations continue to centre on decision-making about CPR. In part, this may be related to consultants' prioritising of ReSPECT conversations with patients for whom CPR would not be medically indicated. As the data were collected at a relatively early stage of ReSPECT implementation, it is also possible that doctors had not yet made the conceptual shift from a DNACPR form to the more holistic approach of the ReSPECT process. Similarly, ReSPECT's key aim – to encourage a patient-centred approach to emergency care treatment planning by prompting patients' explicit involvement in the discussion – was not often realised. This was exemplified by the finding that many of the participating consultants used ReSPECT conversations to inform patients or their relatives about a clinical decision, or to steer them toward a particular decision, rather than engage them in a more open-ended discussion of their wishes and preferences. Moreover, the consultants' focus on patients for whom treatment escalation was not medically indicated also means that other patients, for whom treatment escalation is medically indicated but who may wish to refuse these treatments, may not be given the opportunity to have their wishes respected. This suggests that, at early stages of implementation, the potential of ReSPECT to provide a more holistic patient-centred approach to decision making had not yet been realised fully.

A particular strength of the analysis is the inclusion of consultants from ten acute care and surgical specialities. This enables the representation of diverse secondary-care environments and clinical attitudes to emergency and advance care planning. Additionally, through its two-stage design, whereby each consultant is first shadowed during a ward round and then interviewed, the analysis



For peer review only - http://bmjopen.bmj.com/site/about/guidelines.xhtml

allows for an in-depth discussion of ReSPECT conversations in relation to cases observed by the researcher, thus grounding the interview in specific and varied case examples. The analysis is limited by its focus on consultants. In both sites, consultants were responsible for signing ReSPECT forms; however, as junior doctors and nurses might take part in ReSPECT conversations, it would have been valuable to include their perspectives and experiences. Finally, as the interviews took place within the first year of ReSPECT implementation in both sites, some findings might reflect experiences related to early implementation.

**Conclusion**

The management of uncertainty about prognoses and patients' emotional reactions is central to secondary-care consultants' experiences of ReSPECT conversations. Time constraints and busy ward environments interweave with uncertainty to influence clinicians' decisions about which ReSPECT conversations to prioritize, as does the need to minimise the distress experienced by patients and their relatives and maximise rapport. While some consultants are using the ReSPECT process to broaden conversations about future emergency care treatment plans, many still focus on the decision regarding cardiopulmonary resuscitation and conversations often focus more on communicating and explaining clinical recommendations to patients and their families rather than exploring the patients' values and preferences to inform the decision. This suggests that the aims of the ReSPECT process are yet to be fully realised. Implementation of the ReSPECT process is still in its relatively early stages and our findings may therefore be useful to clinicians and organisations implementing ReSPECT, for example, through informing training on how to conduct ReSPECT conversations while facing uncertainty. Further research should explore how clinicians communicate uncertainty, how patients and families experience uncertainty, and how clinicians' experiences of uncertainty relate to the words and values they employ in engaging in the ReSPECT process.

For peer review only



For peer review only - http://bmjopen.bmj.com/site/about/guidelines.xhtml

## References


1. Resuscitation Council UK. Decisions relating to Cardiopulmonary Resuscitation (3rd edition). 2014. https://www.resus.org.uk/dnacpr/decisions-relating-to-cpr/.

2. General Medical Council. Treatment and care towards the end of life. 2010.

3. Perkins GD. Do Not Attempt Cardiopulmonary Resusitation (DNACPR) Decisions: Evidence Synthesis. Health Services and Delivery Research. NIHR Journals Library. 2016;4(11).

4. Mockford C, Fritz Z, George R, Court R, Grove A, Clarke B, Field R, Perkins GD. Do not attempt cardiopulmonary resuscitation (DNACPR) orders: A systematic review of the barriers and facilitators of decision-making and implementation. Resuscitation 2015;88C:99-113.

5. Freeman K, Field RA, Perkins GD. Variation in local trust Do Not Attempt Cardiopulmonary Resuscitation (DNACPR) policies: a review of 48 English healthcare trusts. BMJ Open 2015;5:e006517.

6. Clements M, Fuld J, Fritz Z. Documentation of resuscitation decision-making: a survey of practice in the United Kingdom. Resuscitation 2014;85:606-11.

7. Fritz Z, Fuld J, Haydock S, Palmer C. Interpretation and intent: a study of the (mis)understanding of DNAR orders in a teaching hospital. Resuscitation 2010;81:1138-41.

8. Findlay GP, Shotton H, Kelly K, Mason M. Time to Intervene? A review of patients who underwent cardiopulmonary resuscitation as a result of an in-hospital cardiorespiratory arrest. A report by the National Confidential Enquiry into Patient Outcome and Death. Dave Terrey; 2012.

9. Cohn S, Fritz ZBM, Frankau JM, Laroche CM, Fuld JP. Do Not Attempt Cardiopulmonary Resuscitation orders in acute medical settings: a qualitative study. QJM 2013;106:165-77.

10. McAdam C, Barton A, Bull P, Rai G. An audit of nurses' views on DNR decisions in 1989 and 2003. *Br J Nurs* 2005;14:1061-2, 4-5.

11. Fritz Z, Cork N, Dodd A, Maylon A. DNACPR decisions: challenging and changing practice in the wake of the Tracey judgment. *Clin Med* 2014;14:571-6.

12. Myint PK, Miles S, Halliday DA, Bowker LK. Experiences and views of specialist registrars in geriatric medicine on 'do not attempt resuscitation' decisions: a sea of uncertainty? QJM 2006;99:691-700.

13. Myint PK, Rivas CA, Bowker LK. In-hospital cardiopulmonary resuscitation: Trainees' worst and most memorable experiences. QJM 2010;103:865-73.

14. NIHR HS&DR. (2016). Evaluation of the Recommended Summary Plan for Emergency Care and Treatment. https://www.journalslibrary.nihr.ac.uk/programmes/hsdr/151509/#/ [accessed 19 February 2019].

15. Fritz Z, Slowther AM, Perkins GD. Resuscitation policy should focus on the patient, not the decision. *BMJ* 2017 28;356:j813.

16. Resuscitation Council UK. 2019. ReSPECT. https://www.resus.org.uk/respect/

17. Czarniawska-Joerges B. Shadowing: and other techniques for doing fieldwork in modern societies. Copenhagen Business School Press DK; 2007.




For peer review only - http://bmjopen.bmj.com/site/about/guidelines.xhtml

18. Davies K. The body and doing gender: the relations between doctors and nurses in hospital work. Sociology of Health & Illness. 2003 Nov;25(7):720-42.

19. Rice T. 'The hallmark of a doctor': the stethoscope and the making of medical identity. Journal of Material Culture. 2010 Sep;15(3):287-301.

20. Braun V, Clarke V. Using thematic analysis in psychology. Qual Res Psychol 2006;3(2):77-101.

21. Tracey, R (On the Application Of) v Cambridge University Hospitals NHS Foundation Trust & Ors 2014;EWCA Civ 822 (17 June 2014) http://www.bailii.org/ew/cases/EWCA/Civ/2014/822.html

22. Holland CL, Bowker LK, Myint PK. Barriers to involving older people in their resuscitation decisions: the primary–secondary care mismatch highlights the potential role of general practitioners. International journal of clinical practice. 2013 Apr;67(4):379-84.

23. Almack K, Cox K, Moghaddam N, Pollock K, Seymour J. After you: conversations between patients and healthcare professionals in planning for end of life care. BMC Palliative Care 2012;11(1):15.

24. Sharp T, Moran E, Kuhn I, Barclay S. Do the elderly have a voice? Advance care planning discussions with frail and older individuals: a systematic literature review and narrative synthesis. *Br J Gen Pract* 2013;63(615):e657-68.

25. Lund S, Richardson A, May C. Barriers to advance care planning at the end of life: an explanatory systematic review of implementation studies. *PLOS ONE* 2015;10:e0116629.

26. Beresford EB. Uncertainty and the shaping of medical decisions. Hastings Center Report 1991 8;21(4):6-11.

27. Griffiths F, Green E, Bendelow G. Health professionals, their medical interventions and uncertainty: A study focusing on women at midlife. *Soc Sci Med* 2006;62(5):1078-90.

28. Griffiths F, Green E, Tsouroufli M. The nature of medical evidence and its inherent uncertainty for the clinical consultation: qualitative study. *BMJ* 2005;330(7490):511.

29. Wray CM, Loo LK. The diagnosis, prognosis, and treatment of medical uncertainty. *J Grad Med Educ* 2015;7:523-527.

30. Ardagh M. Futility has no utility in resuscitation medicine. Journal of medical ethics. 2000 Oct 1;26(5):396-9.

For peer review only



For peer review only - http://bmjopen.bmj.com/site/about/guidelines.xhtml

**Footnotes**

Patient consent for publication: Not required

Contributors: KE conducted data analysis and drafted the manuscript. AMS, FG and CH designed the study, supervised the data collection, contributed to data analysis and commented on manuscript drafts. GDP and KC contributed to study design, assisted with accessing the field and commented on manuscript drafts. CO collected the data and commented on manuscript drafts. All authors reviewed and approved the final version of the manuscript.

Funding: This article presents independent research funded by the National Institute for Health Research (NIHR) under the Health Services and Delivery Research programme (project number 15/15/09). The views expressed in this publication are those of the authors and not necessarily those of the NIHR or the Department of Health and Social Care. KC is supported by an NIHR post-doctoral research fellowship.

Competing interests: CH is a member of the ReSPECT national working group and was involved in the evaluation of ReSPECT. GDP is a member of the ReSPECT national working group and held a leading role in the development of ReSPECT; however, GDP was not involved in data collection or analysis related to the present study. AS, FG, CH, KC and GDP received grants from the UK National Institute of Health Research during the study.

Ethics approval: The study received ethics approval from the NRES Committee, West Midlands – Coventry and Warwickshire (REC reference: 17/WM/0134).

Data sharing statement: Although the qualitative data in this study have been pseudonimised, it is possible that with access to raw data individuals might be identifiable. The data are not suitable for sharing beyond what is contained within the manuscript. Further information can be obtained from the corresponding author.



For peer review only - http://bmjopen.bmj.com/site/about/guidelines.xhtml

# Reporting checklist for qualitative study.

Based on the SRQR guidelines.

## Instructions to authors

Complete this checklist by entering the page numbers from your manuscript where readers will find each of the items listed below.

Your article may not currently address all the items on the checklist. Please modify your text to include the missing information. If you are certain that an item does not apply, please write "n/a" and provide a short explanation.

Upload your completed checklist as an extra file when you submit to a journal.

In your methods section, say that you used the SRQRreporting guidelines, and cite them as:

O'Brien BC, Harris IB, Beckman TJ, Reed DA, Cook DA. Standards for reporting qualitative research: a synthesis of recommendations. Acad Med. 2014;89(9):1245-1251.

| | | Reporting Item | Page Number |
|---|---|---|---|
| **Title** | | | |
| | #1 | Concise description of the nature and topic of the study identifying the study as qualitative or indicating the approach (e.g. ethnography, grounded theory) or data collection methods (e.g. interview, focus group) is recommended | 1 |

For peer review only

## Abstract

| | | | |
|---|---|---|---|
| | #2 | Summary of the key elements of the study using the abstract format of the intended publication; typically includes background, purpose, methods, results and conclusions | 1 |

## Introduction

| | | | |
|---|---|---|---|
| Problem formulation | #3 | Description and signifcance of the problem / phenomenon studied: review of relevant theory and empirical work; problem statement | 3 |
| Purpose or research question | #4 | Purpose of the study and specific objectives or questions | 3-4 |

## Methods

| | | | |
|---|---|---|---|
| Qualitative approach and research paradigm | #5 | Qualitative approach (e.g. ethnography, grounded theory, case study, phenomenolgy, narrative research) and guiding theory if appropriate; identifying the research paradigm (e.g. postpositivist, constructivist / interpretivist) is also recommended; rationale. The rationale should briefly discuss the justification for choosing that theory, approach, method or technique rather than other options available; the assumptions and limitations implicit in those choices and how those choices influence study conclusions and transferability. As appropriate the rationale for several items might be | 4 |

For peer review only

| | | | |
|---|---|---|---|
| | | discussed together. | |
| Researcher characteristics and reflexivity | #6 | Researchers' characteristics that may influence the research, including personal attributes, qualifications / experience, relationship with participants, assumptions and / or presuppositions; potential or actual interaction between researchers' characteristics and the research questions, approach, methods, results and / or transferability | 4 |
| Context | #7 | Setting / site and salient contextual factors; rationale | 4 |
| Sampling strategy | #8 | How and why research participants, documents, or events were selected; criteria for deciding when no further sampling was necessary (e.g. sampling saturation); rationale | 4 |
| Ethical issues pertaining to human subjects | #9 | Documentation of approval by an appropriate ethics review board and participant consent, or explanation for lack thereof; other confidentiality and data security issues | 19 |
| Data collection methods | #10 | Types of data collected; details of data collection procedures including (as appropriate) start and stop dates of data collection and analysis, iterative process, triangulation of sources / methods, and modification of procedures in response to evolving study findings; rationale | 4 |
| Data collection | #11 | Description of instruments (e.g. interview guides, | 4 |

For peer review only

| | | | |
|---|---|---|---|
| instruments and technologies | | questionnaires) and devices (e.g. audio recorders) used for data collection; if / how the instruments(s) changed over the course of the study | |
| Units of study | #12 | Number and relevant characteristics of participants, documents, or events included in the study; level of participation (could be reported in results) | 4 |
| Data processing | #13 | Methods for processing data prior to and during analysis, including transcription, data entry, data management and security, verification of data integrity, data coding, and anonymisation / deidentification of excerpts | 4-5 |
| Data analysis | #14 | Process by which inferences, themes, etc. were identified and developed, including the researchers involved in data analysis; usually references a specific paradigm or approach; rationale | 4-5 |
| Techniques to enhance trustworthiness | #15 | Techniques to enhance trustworthiness and credibility of data analysis (e.g. member checking, audit trail, triangulation); rationale | 4-5 |
| **Results/findings** | | | |
| Syntheses and interpretation | #16 | Main findings (e.g. interpretations, inferences, and themes); might include development of a theory or model, or integration with prior research or theory | 5-14 |
| Links to empirical data | #17 | Evidence (e.g. quotes, field notes, text excerpts, photographs) to substantiate analytic findings | 5-14 |

For peer review only

## Discussion

| | | | |
|---|---|---|---|
| Intergration with prior work, implications, transferability and contribution(s) to the field | #18 | Short summary of main findings; explanation of how findings and conclusions connect to, support, elaborate on, or challenge conclusions of earlier scholarship; discussion of scope of application / generalizability; identification of unique contributions(s) to scholarship in a discipline or field | 14-16 |
| Limitations | #19 | Trustworthiness and limitations of findings | 15-16 |

## Other

| | | | |
|---|---|---|---|
| Conflicts of interest | #20 | Potential sources of influence of perceived influence on study conduct and conclusions; how these were managed | 19 |
| Funding | #21 | Sources of funding and other support; role of funders in data collection, interpretation and reporting | 19 |

The SRQR checklist is distributed with permission of Wolters Kluwer © 2014 by the Association of American Medical Colleges. This checklist was completed on 13. May 2019 using https://www.goodreports.org/, a tool made by the EQUATOR Network in collaboration with Penelope.ai

For peer review only

BMJ Open

# Secondary Care Consultant Clinicians' Experiences of Conducting Emergency Care and Treatment Planning Conversations in England: An Interview-Based Analysis

| | |
|---|---|
| Journal: | *BMJ Open* |
| Manuscript ID | bmjopen-2019-031633.R2 |
| Article Type: | Original research |
| Date Submitted by the Author: | 03-Oct-2019 |
| Complete List of Authors: | Eli, Karin; University of Warwick, Division of Health Sciences, Warwick Medical School<br>Ochieng, Cynthia; Cardiff University, School of Medicine<br>Hawkes, Claire; University of Warwick Warwick Medical School, Clinical Trials Unit<br>Perkins, Gavin; University of Warwick, Clinical Trials Unit<br>Couper, Keith; University of Warwick, Warwick Medical School<br>Griffiths, Frances; University of Warwick, Warwick Medical School<br>Slowther, Anne-Marie; University of Warwick, Warwick Medical School |
| <b>Primary Subject Heading</b>: | Health services research |
| Secondary Subject Heading: | Patient-centred medicine, Qualitative research |
| Keywords: | Emergency care treatment planning, Advance care planning, Clinicians' experiences, Do not attempt cardiopulmonary resuscitation (DNACPR), QUALITATIVE RESEARCH, Decision making |
| | |

SCHOLARONE™
Manuscripts

# Secondary Care Consultant Clinicians' Experiences of Conducting Emergency Care and Treatment Planning Conversations in England: An Interview-Based Analysis

Karin Eli[1], Cynthia Ochieng[2], Claire Hawkes[1], Gavin D. Perkins[1, 3], Keith Couper[1, 3], Frances Griffiths[1]*, Anne-Marie Slowther[1]*

[1] Warwick Medical School, University of Warwick

[2] School of Medicine, Cardiff University

[3] Critical Care Unit, University Hospitals Birmingham NHS Foundation Trust, Birmingham

*These authors share senior authorship

Corresponding author: Karin Eli (Karin.Eli@warwick.ac.uk)

For peer review only

**Abstract**

Objective: To examine secondary-care clinicians' experiences of conducting conversations about treatment escalation with patients and their relatives, using the Recommended Summary Plan for Emergency Care and Treatment (ReSPECT) process.

Design: Semi-structured interviews following ward round observations.

Setting: Two National Health Service hospitals in England.

Participants: Fifteen medical and surgical consultants from 10 specialties, observed in 14 wards.

Analysis: Interview transcripts were analysed using thematic analysis.

Results: Three themes were developed: (1) Determining when and with whom to conduct a ReSPECT conversation; (2) Framing the ReSPECT conversation to manage emotions and relationships; and (3) Reaching ReSPECT decisions. The results showed that when timing ReSPECT conversations, clinicians rely on their predictions of a patient's short-term prognosis; when framing ReSPECT conversations, clinicians seek to minimize distress and maximize rapport; and when involving a patient or a patient's relatives in decision-making discussions, clinicians are guided by their level of certainty about the patient's illness trajectory.

Conclusions: The management of uncertainty about prognoses and about patients' emotional reactions is central to secondary-care clinicians' experiences of timing and conducting ReSPECT conversations.

**Keywords**

Emergency care treatment planning; advance care planning; clinicians' experiences; critical care; do not attempt cardiopulmonary resuscitation (DNACPR); decision making; qualitative research; recommended summary plan for emergency care and treatment (ReSPECT); uncertainty.

**Article summary: strengths and limitations of this study**

- Data were collected from clinicians from ten medical specialities, thereby representing diverse secondary-care environments and clinical attitudes to emergency care and treatment planning.



For peer review only - http://bmjopen.bmj.com/site/about/guidelines.xhtml

- Each clinician was first shadowed during a ward round and then interviewed, thus grounding the interviews in specific and varied case examples.
- The findings reported in this paper are limited by the study's focus on consultants' interviews; as other members of multidisciplinary teams also participate in ReSPECT conversations, including their perspectives and experiences would have been valuable.
- The interviews took place within the first year of ReSPECT implementation in the two study sites, such that some findings may reflect experiences related to early implementation.

For peer review only



For peer review only - http://bmjopen.bmj.com/site/about/guidelines.xhtml

**Introduction**

UK clinical practice guidelines indicate that cardiopulmonary resuscitation (CPR) may be withheld when clinicians predict it would not succeed, if the patient refuses CPR, or following careful clinical assessment of the benefits and burdens of CPR.[1 2] While Do Not Attempt Cardiopulmonary Resuscitation (DNACPR) guidelines are clearly articulated, several studies and reviews have found that, in practice, DNACPR processes are fraught with ambiguity. Clinicians have varying, sometimes-divergent understandings of DNACPR decision-making processes, leading to inconsistencies in how decisions are made, implemented, and recorded.[3-6] These inconsistencies may lead to lower quality of care; indeed, some clinicians misinterpret DNACPR decisions as limiting other aspects of treatment, while others administer CPR inappropriately, failing to follow patients' wishes for the withholding of resuscitation.[3 7-10] Notably, clinicians often communicate poorly about DNACPR with patients and their relatives, and some are reluctant to discuss resuscitation, thereby excluding patients from the decision-making process.[4 6 7 11-13]

This paper is part of a larger study, funded by the National Institute for Health Research (NIHR), which evaluates the Recommended Summary Plan for Emergency Care and Treatment (ReSPECT).[14] Launched in 2017 across NHS Trusts, ReSPECT is an emergency care treatment plan (ECTP) developed in response to the gaps observed in the DNACPR process. ReSPECT builds on research conducted in the US, the UK, and Canada, which found that programmes that integrate DNACPR with discussions about wider goals of treatment increase clarity about trajectories of care and reduce harm to patients.[15] As an ECTP which records clinical recommendations that take into account patients' values and preferences, ReSPECT places resuscitation within a wider context of treatments that should or should not be considered in an emergency situation.

The authors of ReSPECT emphasise that it is a process designed to guide clinicians in discussing with patients what might be optimal treatment choices for them with the ReSPECT form acting as a prompt and summary record of the discussion and its outcomes.[16] The form and its associated guidance documents were developed in 2016 by the ReSPECT working group. Chaired by the Resuscitation Council (UK) and Royal College of Nursing, the ReSPECT working group had representation from patients, professional organisations (Royal Colleges, British Medical Association) regulatory bodies (General Medical Council, Nursing Midwifery Council), the Care Quality Commission, NHS organisations (Acute, Community and Ambulance Trusts) and patient and public members. The completed ReSPECT form is held by the patient, allowing them to communicate the treatment plans when they move from one healthcare setting to another.

In the present paper, we report findings from interviews with secondary-care consultant clinicians in two NHS organisations that had recently implemented ReSPECT, exploring why, when, and with whom they choose to have ReSPECT conversations. Our aim is to inform future development of the process and the current implementation across the NHS and to provide focus to further qualitative research on how ReSPECT becomes integrated into health professionals' practice.



For peer review only - http://bmjopen.bmj.com/site/about/guidelines.xhtml

**Methods**

Fifteen consultants (six female, nine male) from two acute NHS teaching hospitals in England were interviewed from August to December 2017 as part of a wider ongoing study, aimed at evaluating the implementation of the ReSPECT process. The 15 consultants represented 14 wards and 10 medical specialities. Interviews took place in site one 7-10 months after ReSPECT had been implemented and in Site two, 11-12 months after implementation. We observed no differences related to ReSPECT implementation timelines between the two sites.

Potential participants were identified by the local principal investigator at each of the participating hospitals through purposive sampling designed to represent a range of views about the ReSPECT process, as well as a diversity of clinical areas that could be replicated across NHS trusts (three medical specialities, a surgical speciality, and orthopaedics). The local PIs or research nurses asked for volunteer participants from these specialities and the study's research fellow scheduled ward round observations directly with the participating consultants, to ensure that observations did not place an undue burden on their clinical practice. All participating consultants provided written informed consent prior to taking part in the study.

The research fellow, a public health researcher, shadowed each consultant during a ward round, to observe when and how consultants engaged in ReSPECT conversations with their patients. Shadowing is a structured observation technique[17] which has been identified as appropriate for qualitative research on clinicians' experiences and practices.[18 19] To ensure that patients, relatives, and staff were aware that observations were taking place, study posters were displayed in the selected wards, and the research fellow wore a scrubs uniform top with the word 'researcher' printed clearly on both the front and the back. During each shadowing period the participating consultant introduced the researcher to each patient (and family if appropriate) and informed them that they could request that the researcher leave if they wished. A brief information leaflet was left with the patient. The researcher interviewed each consultant following the observation, typically within 24 -48 hours. The interviews were semi-structured and were designed to explore each consultant's decision-making about holding a ReSPECT conversation in three observed cases, as well as the consultant's wider experiences with ReSPECT. If the researcher observed three ReSPECT conversations (which was the maximum she had observed in any of her observation sessions), she selected these three cases for discussion during the interviews. If she observed fewer than three ReSPECT conversations, she selected one or more cases where she thought a ReSPECT conversation might have been appropriate, to explore with the clinician why they chose not to hold a ReSPECT conversation in those cases. The interview topic areas were developed based on the study's research questions and the literature, and the observation and interview approach was checked with members of the study team with relevant clinical experience. The interviews lasted from 15 to 53 minutes, with a median time of 37 minutes, and were digitally recorded and transcribed.

Interview transcripts were analysed by the study's senior research fellow (SRF), a medical anthropologist, using thematic analysis.[20] First, the SRF read the interview transcripts to identify initial codes. The transcripts were then coded closely, with most codes developed at the level of sentences or sentence clauses. The SRF reviewed the coded interviews, and grouped the codes to develop themes. The themes were continuously revised throughout the process of reviewing the coded interviews, leading to 16 emerging themes, which were grouped into overarching themes. To



For peer review only - http://bmjopen.bmj.com/site/about/guidelines.xhtml

ensure intercoder reliability, four of the 15 interviews were analysed independently by another SRF, a health services researcher. The two SRFs discussed the codes, identified differences and potential disagreements, and discussed these until they reached consensus. In total, five initial overarching themes were developed: three focused on the ReSPECT conversation, one focused on consultants' value judgments, and one focused on the ReSPECT form. After they achieved consensus, the SRFs discussed the five overarching themes with two of the senior co-authors, doctors with research expertise in medical ethics and medical sociology. Together, they decided to focus the analysis on the three overarching themes concerned with the ReSPECT conversation, as these themes most closely responded to the study's aim of exploring why, when, and with whom consultants choose to have ReSPECT conversations. Finally, the first SRF reviewed all interview transcripts to ensure the three themes represented the data accurately. Throughout the analytic process, coding was conducted using word processing software. To maintain participant confidentiality, the gender-neutral pronouns they/them are used to refer to all consultants.

<u>Patient and public involvement (PPI)</u>

The study has been supported throughout by a PPI group, which informed the study design and the development of models of recruitment and consent. Additionally, PPI group members provided feedback on a draft of the manuscript. They agreed with the manuscript's findings and offered suggestions for areas to investigate further in our future research, in light of their own experiences as patients or carers.

## Results

<u>*Theme 1: Determining when and with whom to conduct a ReSPECT conversation: Uncertainty management and catalysts for discussion*</u>

Given time constraints, consultants had to determine which patients were most in need of a ReSPECT conversation, and when this conversation should be conducted. Making these determinations was fraught with uncertainty. To manage this uncertainty, consultants relied on their predictions and imaginings of patients' immediate futures, using the ward round to piece together prognostic puzzles. For example, explaining why he did not initiate a ReSPECT conversation with a patient in her 90s, this consultant said:

> *she's otherwise recovering well (…) I thought the chances of her, as it were, needing any of the interventions you might discuss on a ReSPECT form were small. (Site 1, C10)*

The key to initiating a ReSPECT conversation, this consultant later explained, was predicting a trajectory of deterioration:

> *It's the deteriorating patients, patients with end-of-life conditions for whatever reason, be it cancer or organ failure and any patient where they might suddenly deteriorate. (Site 1, C10)*

Another consultant said they used the ward round to predict whether a patient was likely to experience a 'catastrophe'; such a prediction, they said, would warrant initiating a ReSPECT conversation:



For peer review only - http://bmjopen.bmj.com/site/about/guidelines.xhtml

*Particularly where you're seeing acutely ill patients and you're seeing them for the first time as, as an acute physician, I think the prompt is how likely you think it is that this patient may have a catastrophe, may have a cardiac arrest, may have a sudden severe deterioration. (Site 1, C11)*

This consultant, like numerous others in the sample, linked the focus on predicted deterioration to the time constraints of the ward round. As another consultant explained:

*I think in the context of a post-take ward round where I am time limited I prioritise those patients for whom these conversations are most likely to be required for this admission. So it may well be that ReSPECT conversations were appropriate for more of the patients that I saw today in terms of potentially being last year of life. But they were not decisions that were required today. (Site 2, C17)*

While time constraints were frequently cited, they were not the only factor underlying consultants' focus on predicted deterioration. Explaining why they were less likely to initiate a ReSPECT conversation with some patients, this consultant positioned their decision-making as culturally embedded:

*I think for the time being the culture is still the ReSPECT form is mainly for when people deteriorate. I think sometimes asking people a hypothetical question when they're really quite well, it's difficult to frame it. (Site 1, C09)*

This consultant linked their focus on deterioration as the primary prompt for a ReSPECT conversation to the difficulty of asking patients to imagine a hypothetical catastrophic scenario. Imagining difficult scenarios, however, was central to ReSPECT conversations. Since initiating ReSPECT conversations depended on clinicians' predictions of patients' short-term prognoses, ReSPECT conversations engaged patients with clinicians' predictive thinking. This process was often challenging, as patients did not necessarily share in the logics and concepts of time posed by their clinicians:

*[P]eople find, "What if?" challenging. So if I say, "What if you're going to deteriorate? We need to make a decision what we would do about ITU." A lot of patients and relatives will hear about us saying, "You're deteriorating, you're going to need ITU." They don't hear the "What if?" (Site 2, C17)*

Like others, this consultant explained that while they framed possible future scenarios in subjunctive – that is, potential or conditional – terms, patients and relatives tended to understand these in definitive future terms. Another consultant explained that, rather than joining a dialogue on potential scenarios, some patients and relatives expressed distress over what they understood as a terminal prognosis:

*…even though I've said "I am going to let you go home now, have you thought about what you would like in the future?", and then they say "why am I going to die? You're telling me I am going to die aren't you!" (Site 1, C01)*



For peer review only - http://bmjopen.bmj.com/site/about/guidelines.xhtml

In addition to predicted deterioration, consultants timed ReSPECT conversations according to calculations of risk related to a wider network of actors. The timing of ReSPECT conversations therefore implicated not only patients, but also other clinicians. For example, some consultants said they were reluctant to hold ReSPECT conversations with pre-operative patients, as these discussions could bias surgeons or demoralise patients:

> *[The patient] was going to undergo an operation and I feel very uncomfortable discussing resuscitation just before the operation (…) if he does not want to be resuscitated, it influences the surgeon to some extent. (Site 2, C14)*

> *…we don't often talk about ReSPECT form because it's, you know, when you deteriorate. And in some ways with elective surgery they're very much focused on consenting for surgery and talking about rehab after surgery rather than deterioration from surgery. (Site 1, C08)*

Elsewhere in the interview, this surgeon said the best time to initiate a ReSPECT conversation was immediately after surgery – a particularly opportune time because, while risk of complications was higher after surgery, patients' distress was likely to be lower, and relatives were likely to be present:

> *…often, often there's a family around at that point because it's usually an emergency admission and the family come in a day or two later. So you can involve the parties that you need to at that point in time. It's a relatively rare event for a patient to die on the operating table, if they're going to, if they're going to succumb it's usually over the following few days. (Site 1, C08)*

The presence of relatives was central to the timing of many ReSPECT conversations, not least because conducting the ReSPECT process with patients who lack capacity requires the participation of an individual close to the patient. In this example, a consultant discussed a case where a visit from a patient's relatives prompted a ReSPECT conversation:

> *[H]is family were there so I took the opportunity while they were all there to express not only that he was perhaps more unwell than they had recognised, and that he was getting better with treatment, and to explore what their feelings were about escalation of care, particularly whether intensive care would be appropriate for him. (Site 2, C17)*

This consultant considered the presence of relatives crucial in timing the ReSPECT conversation due to uncertainty about escalating the patient's care. Other consultants, however, spoke of the presence of relatives as important for finalizing and communicating a medical decision, rather than deliberating about a trajectory of care.

*<u>Theme 2: Conducting the ReSPECT conversation: Managing emotions and relationships</u>*

Most ReSPECT conversations implicated a triad of patient, clinician(s), and relative(s). For patients who lacked capacity, this triad was essential to the ReSPECT process, with relatives or other advocates called upon to speak on the patient's behalf. However, while patients with capacity could speak privately with their clinicians, they often involved their relatives, framing the ReSPECT



For peer review only - http://bmjopen.bmj.com/site/about/guidelines.xhtml

conversation and their own decision-making as familial. In these cases, consultants clarified they included relatives in the conversation, but did not involve them in decision-making:

> *…you've had a discussion, the patient says, "Look, I don't want anything done, doctor," I think it's still very valuable to the next of kin to know that. (…) but we're not asking the family to participate in the discussion if the patient has already made their wishes clear in a reliable way. (Site 1, C11)*

Although consultants tended to describe relatives' involvement as valuable, they also described it as potentially problematic, characterising family members as either compliant and 'sensible', or as non-compliant and 'difficult'. According to some consultants, relatives sometimes challenged clinical decisions – specifically, decisions against resuscitation – because they misunderstood what 'not for resuscitation' meant for the patient's future care:

> *Sometimes you have relatives who are very emotional, sometimes they think when you say 'not for resuscitation' means you're going to stop all treatment. (Site 2, C16)*

In other cases, consultants said relatives misunderstood their role in the ReSPECT conversation as that of 'decision-maker', worrying about how a 'not for resuscitation' decision might reflect on them:

> *Often what happens is the relatives feel that you're asking them to make the decision… and again because they've been misled by the media, they feel that if they say, yes, make them not for resuscitation, that they might be seen as a money grabbing. (Site 2, C12)*

Disagreement between clinicians and patients' relatives could carry consequences for patient care, particularly if relatives who held legal power of attorney (LPA) attempted to overturn a clinical decision. In those cases, consultants advocated for their clinical decision, taking the role of acting on the patient's behalf:

> *I try to explain to them that by keeping them alive, you are, you are, you are prolonging their agony. (…) I try to avoid confrontation with them (…) But sometimes we have to, when I can see clearly that there is going to be harm, then I have to, even if they have the LPA. (Site 2, C14)*

Another relational aspect of the ReSPECT conversation was the consideration of other, sometimes absent, clinicians. Several consultants spoke about the importance of identifying the 'right' clinician to conduct a ReSPECT conversation – often, the consultant or the GP regularly charged with the patient's care. In post-take ward rounds, some consultants avoided conducting ReSPECT conversations with patients who were usually seen by their colleagues. In this example, a consultant explained why they chose not to complete a ReSPECT form with a patient who had a localised infection:

> *I could've completed a ReSPECT form but I didn't because I, effectively I'm not looking at her [as her] responsible consultant. (Site 2, C12)*



For peer review only - http://bmjopen.bmj.com/site/about/guidelines.xhtml

Later in the interview, this consultant explained that the patient's condition did not warrant an urgent ReSPECT conversation. Given the lack of urgency, they deferred to the patient's 'usual physicians, who obviously know her prognosis'. It would be inappropriate, this consultant argued, to conduct a ReSPECT conversation with a patient whose consultants evidently did not deem it necessary.

Consultants deferred ReSPECT conversations until the 'right' clinicians could conduct them not only because patients' usual consultants were more knowledgeable about these patients' medical histories, but also because these usual consultants had established rapport with the patients. In this example, a consultant explained why they chose to conduct a ReSPECT conversation but leave the final decision for a future discussion between the patient and her usual consultant. The patient, this consultant explained, was not 'receptive' to an earlier ReSPECT conversation with her usual consultant. As such, they viewed their role as providing a second opinion to support the consultant's, rather than as finalizing a ReSPECT decision.

> *…I didn't feel as if I was going to be welcomed to take that further with her myself. So I thought it was better than to say, to see her back to her normal consultant then the next time. (Site 2, C13)*

For similar reasons, other consultants suggested that ReSPECT conversations were best conducted in primary care settings, led by patients' GPs rather than by clinicians they first met during an acute care admission. In response to the researcher's question, 'So you think this is something that should be discussed in the community?', this consultant said:

> *Definitely because I think it makes… patients feel less vulnerable… when they are in hospital they feel vulnerable plus they don't know us (…) they might have known the GP or have some sort of on-going or community matron or something that's a bit more of a long term relationship. (Site 1, C04)*

The importance consultants placed on rapport was closely connected to their concerns over trustworthiness. Worries about being perceived as untrustworthy led some consultants to avoid or delay ReSPECT conversations with some patients. As described by consultants, ReSPECT conversations, if not framed carefully, could undermine the process of building trust with patients.

> *One of my worries is that patients, if you're not careful with your language, a patient might interpret a discussion about what to do in the event of deterioration, escalation, CPR, etcetera, as you giving up on them, as you not being prepared to do everything that you can to get them over their illness. (Site 1, C11)*

The timing of ReSPECT conversations could also affect trust building. This consultant, for example, suggested that initiating a ReSPECT conversation too early would shake the patient's trust:

> *… you want to make sure you still have the rapport with the patient, that they see you as somebody that's there to help them (…) and if you feel that the patient is not quite ready to*



For peer review only - http://bmjopen.bmj.com/site/about/guidelines.xhtml

> *talk about it or they don't want to then if you kind of push it they'll see you negatively. (Site 1, C04)*

Another consultant, a surgeon, described a case where they conducted a ReSPECT conversation before operating on a seriously ill patient. While the ReSPECT conversation was carefully timed from a medical perspective, it forced the patient to confront difficult scenarios that destabilized her trust in the surgeon:

> *So she doesn't want to talk about whether or not she's going to die on the operating table, or whether or not she's going to get her post-op chest infection or a lung embolus or whatever else could happen. But that process makes us talk about it at that point in time. (…) as soon as you mention that sentence about what would you like to do and if things were taking a turn for the worse (…) she's switched, she's completely switched off. (Site 1, C08)*

Notably, consultants were concerned about being perceived as trustworthy because they identified the ReSPECT conversation as a catalyst for potential distress for patients and relatives. To manage the difficult emotions that often arose during ReSPECT conversations, consultants used various techniques: from avoiding the conversation if the patient was expected to react aggressively or become overwhelmed, to initiating a series of conversations to ease patients and relatives into their future trajectory. For example, one consultant deferred ReSPECT conversations with patients recently diagnosed with terminal cancer to avoid overwhelming them:

> *I've generally just told them they've got incurable cancer and it, to go on straight from that to a ReSPECT conversation is too much. But I will say that it exists and that it may be something they want to consider and then ask somebody else to follow it up. (Site 2, C17)*

Alongside concerns over patients' emotional wellbeing, several consultants said that previous experiences with patients or relatives who became upset made them cautious about initiating and framing ReSPECT conversations. One consultant, who explained that 'we worry about the angry and anxious one[s]' (Site 2, C17), described beginning each ReSPECT conversation by framing it as common and routine, to pre-empt patients' upset reactions. Another consultant, who described ReSPECT conversations as 'emotionally very draining', conducted repeated ReSPECT conversations to manage relatives' distress:

> *So if you can get some background knowledge, and if they are so in shock that they can't take anything in then it's okay to come back another time. (…) I would prefer to sit away in a, in a room together with a nurse accompanying me, so that you've got a bit of time to yourself and make sure that they know you've got time to listen to them and questions and things. (Site 1, C09).*

The availability of sufficient time and adequate space influenced consultants' capacity to conduct ReSPECT conversations. Many conversations, of necessity, took place during ward rounds, and the crowdedness, urgent pace, and lack of privacy in acute wards limited clinicians' ability to conduct the in-depth ReSPECT conversations they envisioned as appropriate. This consultant, for example,



For peer review only - http://bmjopen.bmj.com/site/about/guidelines.xhtml

argued that ReSPECT conversations necessitated the quiet environment of the patient's home or GP surgery:

> *…this is quite a serious and significant discussion that should not take place in a very busy, busy place. It should either happen when the patient is comfortable in their own home, or, or they have gone to see their, gone to see their GP… (Site 2, C14)*

Hectic ward environments, in this consultant's experience, implicated an urgency and sensory onslaught that, together with patients' acute conditions, led to compromised conversations. The lack of sufficient time to conduct ReSPECT conversations in acute care wards was a pervasive concern across the sample:

> *…it takes time and it sort of stirs up emotions both in you and in the patient (…) so it can be very difficult, mmm, not least because you want to do it well and yet you know we were on a ward round which isn't an ideal kind of, ideally you'd come back and spend 20 minutes with each of them wouldn't you and their families and talk to them at some length. (Site 1, C06)*

Notably, this consultant suggested that lack of time was not simply a logistical issue, but a factor that reduced the ability to conduct careful ReSPECT conversations and manage the emotions that arose during ReSPECT conversations.

*Theme 3: Reaching ReSPECT decisions: Involving versus informing*

The extent to which ReSPECT conversations engaged with patients' wishes depended on consultants' clarity or uncertainty about patients' trajectories. When consultants had clear predictions for patients' short-term prognoses, they tended to lead ReSPECT conversations, taking an informative and persuasive stance. For example, when asked by the researcher, 'Are there times when you find yourself pushing the discussion in a particular way?', this consultant responded:

> *Yes, I think if you genuinely feel that it would be completely futile and that you would only be prolonging an unpleasant death then yes, you do, you do tend to push the discussion in one way or another. (Site 1, C10)*

Consultants often used words such as 'futile', 'frail', or 'co-morbid' when describing cases in which they took a persuasive stance. Futility, as consultants framed it, foreclosed discussion of patient preferences. The conversation focused on patient preferences only when consultants were uncertain about a patient's trajectory:

> *I think that the times where it's very important to discuss with a patient whether they would be appropriate for resuscitation is if it's a patient that maybe is potentially a candidate for intensive care, Level 3 care, that isn't so frail and co-morbid that we feel it would be utterly futile. (Site 2, C12)*

Because they approached ReSPECT conversations according to perceptions of prognostic clarity and uncertainty, many consultants described the ReSPECT conversations in which they typically engaged



For peer review only - http://bmjopen.bmj.com/site/about/guidelines.xhtml

– conversations with patients at imminent risk – as processes of navigation and persuasion. For example, one consultant described handling a patient's son's concerns by 'steer[ing]' the conversation:

> *I went in with quite clear views of what had to be done and as you say the patient's son started to suggest that 'actually he would want to be resuscitated wouldn't you Dad' mmm… and I gently had to steer him away to explain why I didn't think that would be a very good idea. (Site 1, C06)*

As described by consultants, the need to persuade some patients and relatives was the main challenge in the ReSPECT process. To foreclose possibilities for disagreement, some consultants described structuring ReSPECT conversations to clarify which medical procedures would be undertaken:

> *I think a general structure is this is what's wrong, this is what we will do and this is what we won't do and if they are going to be relevant things like feeding, normal ITU, critical care I think these things need to be discussed. (Site 1, C05)*

As this consultant explained, while they clarified that medical decisions were not open for discussion, they attempted to elicit patients' views during the ReSPECT conversation and integrate these into their decision-making processes:

> *when we are looking at what I think we can do medically we have to take into account what the patient believes [and] how they live their life… (Site 1, C05)*

In line with structuring conversations to foreclose debate about medical decisions, some consultants described the ReSPECT conversation as centrally concerned with informing patients and relatives, rather than involving them in medical decision-making. For example, this consultant used the word 'disclose' to describe the function of ReSPECT conversations:

> *I still believe it's a medical decision and it's a good practice to inform the patient and their family. So, ultimately, the decision is mine, but I have to disclose my decision to the patient and their family. (Site 2, C15)*

In other interviews, consultants suggested that, as part of the ReSPECT conversation, clinicians should state explicitly that they are informing patients and relatives about a medical decision, rather than seeking their opinion or approval. One consultant, for example, said that, when conducting a ReSPECT conversation with the relatives of a patient without capacity, one must clarify the relatives' role is to provide contextualising information and ask questions, rather than be actively involved in decision-making:

> *I think doctors in particular need to be clear, they're not handing over the decision making to a family member, they are still responsible for the decision but they're ensuring it's made, as far as possible, in line with what the patient would want. (Site 1 C11)*



For peer review only - http://bmjopen.bmj.com/site/about/guidelines.xhtml

For peer review only

Another consultant said the ReSPECT form itself, in providing space for patient input, needed to be mediated with care, to avoid conveying that medical decisions required relatives' approval:

> *I will normally say that the final decision is a medical decision… 'cause the relatives say 'oh you know I need to check with my brother' when I said that 'I am informing you and just making sure you aware that this is the reason why we are doing it'. (Site 1 C04)*

Along similar lines, a consultant suggested that foregrounding patient views in the ReSPECT conversation was potentially detrimental, as it could place an undue burden on patients or lead to false hope:

> *If it's bleeding obvious what can and can't be offered medically then, then you have to be really careful about getting the patient to express about what they want. (…) It has the ironic effect of making them feel more ignored than they would be if, if you just gently explained what is and isn't possible. (Site 1, CO6)*

According to this consultant, asking patients to express their wishes unreservedly was counterproductive. Instead, this consultant argued, doctors should clarify medical possibilities and impossibilities, not place patients in the vulnerable position of having their wishes denied and their hopes deflated.

Consultants cited clear and careful communication about the finality of medical decisions as a source of comfort to patients. Describing how they would structure a ReSPECT conversation, one consultant related a hypothetical scenario in which an 82-year-old patient was diagnosed with terminal cancer. In this scenario, they said, they would relate the news to the patient, cite the evidence (as provided by blood tests), and explain what treatments will and will not be offered. Using the second person singular, the consultant described what they would say to this hypothetical patient:

> *"Our aim will be to keep you comfortable, to support you through this. If you have any pain we will, we will control it with strong painkillers. If you have any sickness we will do that. If the time comes and if you stop breathing, or if your heart stops pumping blood… we will not be doing resuscitations, or we will not jump on your chest and perform cardiac compressions because it's not going to work. We will let you go in dignity and respect, and we will support you in that process. We will make sure your family's around you if we can." (Site 2, C16)*

Reflecting on this scenario, they said this approach 'reassured' patients:

> *…if you're very clear to them then they can decide whether they want to be at home, whether they want to be in the hospital. And it just helps them. And if you're quite open to them, they will openly ask you questions and it just makes things easy. (Site 2, C16)*

While most consultants shared a directive approach to the ReSPECT conversation, particularly in cases where they deemed resuscitation 'futile', it was not the default option for all. One consultant, for example, conceptualized the ReSPECT conversation as 'patient-centred' and as a dialogic process toward a shared decision:

*So you start off by, by getting the patient to, to give their thoughts on what they would or wouldn't like. And that allows you to, to guide the final decision. Perhaps that's not, so it's not necessarily the patient starting with it. But you do it together. (Site 2, C13)*

Framing the ReSPECT conversation as a dialogue did not preclude medical decision-making. Elsewhere in the interview, this consultant said they initiated ReSPECT conversations with patients they thought should not be for resuscitation. However, this consultant understood the ReSPECT process as complex, often comprised of multiple conversations with clinical and familial actors, building up to a shared decision. This process, they explained, led to deeper understanding and decisions that empowered patients, especially those who decided to forgo future critical care interventions.

## Discussion

Our analysis found that the management of uncertainty about prognoses and patients' and relatives' emotional reactions is central to consultants' experiences of ReSPECT conversations. When determining when and with whom to conduct ReSPECT conversations, consultants rely on their predictions of a patient's short-term prognosis, prioritising patients for whom they are certain treatment escalation would not be medically indicated. When patients lack capacity, consultants also time conversations to coincide with the presence of patients' relatives, underscoring the importance of involving next of kin in these conversations, as specified in English law.[21] When determining which clinician should conduct a ReSPECT conversation and how the conversation should be framed, consultants seek to maximize rapport and minimize distress, sometimes avoiding or deferring conversations to manage uncertainty about patients' and relatives' emotional reactions. When deciding whether ReSPECT conversations should inform patients about a clinical decision or involve them in decision-making, consultants rely on their clarity or uncertainty about patients' trajectories. Thus, consultants' decisions about with whom to have RESPECT conversations, when to have these conversations, and whether to frame these conversations as explaining medical decisions or as eliciting patients' preferences are driven by consultants' degrees of uncertainty about prognoses, reactions, and outcomes. Throughout, the time-pressured and busy environments of acute care wards influence consultants' decisions about which conversations to prioritise and their experiences of rapport with patients.

Many of the findings are consistent with earlier studies on clinicians' experiences of barriers to DNACPR[10 22] and ACP processes.[23 24] Notably, the findings resonate with a recent systematic review of qualitative studies on the implementation of ACPs, which found that clinicians' uncertainty about prognoses, uncertainty about patients' and relatives' reactions to ACP, and structural constraints related to the clinical environment all constituted barriers to ACP processes.[25] The finding of the central role of uncertainty in ReSPECT conversations both resonates with and diverges from previous research in ways that implicate features particular to ECTPs. Earlier studies have found that negotiating uncertainty is central to medical decision-making and clinical care, particularly when clinicians translate complex population-level evidence to individual prognosis and treatment.[26 27] Nonetheless, when communicating with patients, clinicians often provide reassurance through discursive modes that convey more certainty than is warranted.[28] The present analysis finds that,



For peer review only - http://bmjopen.bmj.com/site/about/guidelines.xhtml

when conducting ReSPECT conversations, particularly with patients whose immediate trajectories are unclear, some consultants present patients and relatives with possible scenarios of future deterioration, to involve them in the decision-making process. Yet these expressions of uncertainty about prognosis and treatment, while consistent with the goals of the ECTP sometimes clash with patients' and relatives' expectations of reassurance, clinical certainty, and definitive knowledge. Previous research has suggested that clinicians can frame expressions of uncertainty productively, as an opening to shared decision-making discussions with patients.[29] Based on the present study's findings, training clinicians in how to frame uncertainty as a conversational prompt may be of particular importance in the implementation of ReSPECT.

Notably, consultants explained how they decide when, with whom, and how to conduct a ReSPECT conversation through keywords which include, among others, 'frail', 'futile', and 'co-morbid'. Such keywords may serve as shorthand for clinicians' ethical stance on trajectories of treatment, although 'frail' and 'co-morbid' may also express clinical assessment. The use of such keywords without reference to clinical assessments may therefore be potentially problematic; 'futility', in particular, has been subject to debate within the medical ethics literature, with some authors arguing that the use of this term, for which no consensus definition exists, can muddle decision-making and hinder patient autonomy.[30] Previous research has found that, on DNACPR forms, clinicians entered keywords such as 'frailty' and 'futility' to justify DNACPR decisions.[9] This analysis suggests that clinicians continue to employ these keywords. How doctors are using these keywords in the context of ReSPECT conversations warrants further exploration.

One aim of the ReSPECT process is to move discussions of future emergency treatment from a focus on CPR to broader considerations of potential treatments. Our analysis shows that some consultants are broadening these discussions. However, in the early adoption phase of ReSPECT, is seems that many conversations continue to centre on decision-making about CPR. In part, this may be related to consultants' prioritising of ReSPECT conversations with patients for whom CPR would not be medically indicated. As the data were collected at a relatively early stage of ReSPECT implementation, it is also possible that doctors had not yet made the conceptual shift from a DNACPR form to the more holistic approach of the ReSPECT process. Similarly, ReSPECT's key aim – to encourage a patient-centred approach to emergency care treatment planning by prompting patients' explicit involvement in the discussion – was not often realised. This was exemplified by the finding that many of the participating consultants used ReSPECT conversations to inform patients or their relatives about a clinical decision, or to steer them toward a particular decision, rather than engage them in a more open-ended discussion of their wishes and preferences. Moreover, the consultants' focus on patients for whom treatment escalation was not medically indicated also means that other patients, for whom treatment escalation is medically indicated but who may wish to refuse these treatments, may not be given the opportunity to have their wishes respected. This suggests that, at early stages of implementation, the potential of ReSPECT to provide a more holistic patient-centred approach to decision making had not yet been realised fully.

A particular strength of the analysis is the inclusion of consultants from ten acute care and surgical specialities. This enables the representation of diverse secondary-care environments and clinical attitudes to emergency and advance care planning. Additionally, through its two-stage design, whereby each consultant is first shadowed during a ward round and then interviewed, the analysis



For peer review only - http://bmjopen.bmj.com/site/about/guidelines.xhtml

allows for an in-depth discussion of ReSPECT conversations in relation to cases observed by the researcher, thus grounding the interview in specific and varied case examples. The analysis is limited by its focus on consultants. In both sites, consultants were responsible for signing ReSPECT forms; however, as junior doctors and nurses might take part in ReSPECT conversations, it would have been valuable to include their perspectives and experiences. Finally, as the interviews took place within the first year of ReSPECT implementation in both sites, some findings might reflect experiences related to early implementation.

**Conclusion**

The management of uncertainty about prognoses and patients' emotional reactions is central to secondary-care consultants' experiences of ReSPECT conversations. Time constraints and busy ward environments interweave with uncertainty to influence clinicians' decisions about which ReSPECT conversations to prioritize, as does the need to minimise the distress experienced by patients and their relatives and maximise rapport. While some consultants are using the ReSPECT process to broaden conversations about future emergency care treatment plans, many still focus on the decision regarding cardiopulmonary resuscitation and conversations often focus more on communicating and explaining clinical recommendations to patients and their families rather than exploring the patients' values and preferences to inform the decision. This suggests that the aims of the ReSPECT process are yet to be fully realised. Implementation of the ReSPECT process is still in its relatively early stages and our findings may therefore be useful to clinicians and organisations implementing ReSPECT, for example, through informing training on how to conduct ReSPECT conversations while facing uncertainty. Further research should explore how clinicians communicate uncertainty, how patients and families experience uncertainty, and how clinicians' experiences of uncertainty relate to the words and values they employ in engaging in the ReSPECT process.

For peer review only



For peer review only - http://bmjopen.bmj.com/site/about/guidelines.xhtml

## References


1. Resuscitation Council UK. Decisions relating to Cardiopulmonary Resuscitation (3rd edition). 2016. https://www.resus.org.uk/dnacpr/decisions-relating-to-cpr/

2. General Medical Council. Treatment and care towards the end of life. 2010.

3. Perkins GD. Do Not Attempt Cardiopulmonary Resusitation (DNACPR) Decisions: Evidence Synthesis. Health Services and Delivery Research. NIHR Journals Library. 2016;4(11).

4. Mockford C, Fritz Z, George R, Court R, Grove A, Clarke B, Field R, Perkins GD. Do not attempt cardiopulmonary resuscitation (DNACPR) orders: A systematic review of the barriers and facilitators of decision-making and implementation. Resuscitation 2015;88C:99-113.

5. Freeman K, Field RA, Perkins GD. Variation in local trust Do Not Attempt Cardiopulmonary Resuscitation (DNACPR) policies: a review of 48 English healthcare trusts. BMJ Open 2015;5:e006517.

6. Clements M, Fuld J, Fritz Z. Documentation of resuscitation decision-making: a survey of practice in the United Kingdom. Resuscitation 2014;85:606-11.

7. Fritz Z, Fuld J, Haydock S, Palmer C. Interpretation and intent: a study of the (mis)understanding of DNAR orders in a teaching hospital. Resuscitation 2010;81:1138-41.

8. Findlay GP, Shotton H, Kelly K, Mason M. Time to Intervene? A review of patients who underwent cardiopulmonary resuscitation as a result of an in-hospital cardiorespiratory arrest. A report by the National Confidential Enquiry into Patient Outcome and Death. Dave Terrey; 2012.

9. Cohn S, Fritz ZBM, Frankau JM, Laroche CM, Fuld JP. Do Not Attempt Cardiopulmonary Resuscitation orders in acute medical settings: a qualitative study. QJM 2013;106:165-77.

10. McAdam C, Barton A, Bull P, Rai G. An audit of nurses' views on DNR decisions in 1989 and 2003. *Br J Nurs* 2005;14:1061-2, 4-5.

11. Fritz Z, Cork N, Dodd A, Maylon A. DNACPR decisions: challenging and changing practice in the wake of the Tracey judgment. *Clin Med* 2014;14:571-6.

12. Myint PK, Miles S, Halliday DA, Bowker LK. Experiences and views of specialist registrars in geriatric medicine on 'do not attempt resuscitation' decisions: a sea of uncertainty? QJM 2006;99:691-700.

13. Myint PK, Rivas CA, Bowker LK. In-hospital cardiopulmonary resuscitation: Trainees' worst and most memorable experiences. QJM 2010;103:865-73.

14. NIHR HS&DR. (2016). Evaluation of the Recommended Summary Plan for Emergency Care and Treatment. https://www.journalslibrary.nihr.ac.uk/programmes/hsdr/151509/#/ [accessed 19 February 2019].

15. Fritz Z, Slowther AM, Perkins GD. Resuscitation policy should focus on the patient, not the decision. *BMJ* 2017 28;356:j813.

16. Resuscitation Council UK. 2019. ReSPECT. https://www.resus.org.uk/respect/

17. Czarniawska-Joerges B. Shadowing: and other techniques for doing fieldwork in modern societies. Copenhagen Business School Press DK; 2007.




For peer review only - http://bmjopen.bmj.com/site/about/guidelines.xhtml

18. Davies K. The body and doing gender: the relations between doctors and nurses in hospital work. Sociology of Health & Illness. 2003 Nov;25(7):720-42.

19. Rice T. ‘The hallmark of a doctor’: the stethoscope and the making of medical identity. Journal of Material Culture. 2010 Sep;15(3):287-301.

20. Braun V, Clarke V. Using thematic analysis in psychology. Qual Res Psychol 2006;3(2):77-101.

21. Tracey, R (On the Application Of) v Cambridge University Hospitals NHS Foundation Trust & Ors 2014;EWCA Civ 822 (17 June 2014) http://www.bailii.org/ew/cases/EWCA/Civ/2014/822.html

22. Holland CL, Bowker LK, Myint PK. Barriers to involving older people in their resuscitation decisions: the primary–secondary care mismatch highlights the potential role of general practitioners. International journal of clinical practice. 2013 Apr;67(4):379-84.

23. Almack K, Cox K, Moghaddam N, Pollock K, Seymour J. After you: conversations between patients and healthcare professionals in planning for end of life care. BMC Palliative Care 2012;11(1):15.

24. Sharp T, Moran E, Kuhn I, Barclay S. Do the elderly have a voice? Advance care planning discussions with frail and older individuals: a systematic literature review and narrative synthesis. *Br J Gen Pract* 2013;63(615):e657-68.

25. Lund S, Richardson A, May C. Barriers to advance care planning at the end of life: an explanatory systematic review of implementation studies. *PLOS ONE* 2015;10:e0116629.

26. Beresford EB. Uncertainty and the shaping of medical decisions. Hastings Center Report 1991 8;21(4):6-11.

27. Griffiths F, Green E, Bendelow G. Health professionals, their medical interventions and uncertainty: A study focusing on women at midlife. *Soc Sci Med* 2006;62(5):1078-90.

28. Griffiths F, Green E, Tsouroufli M. The nature of medical evidence and its inherent uncertainty for the clinical consultation: qualitative study. *BMJ* 2005;330(7490):511.

29. Wray CM, Loo LK. The diagnosis, prognosis, and treatment of medical uncertainty. *J Grad Med Educ* 2015;7:523-527.

30. Ardagh M. Futility has no utility in resuscitation medicine. Journal of medical ethics. 2000 Oct 1;26(5):396-9.

For peer review only

**Footnotes**

Patient consent for publication: Not required

Contributors: KE conducted data analysis and drafted the manuscript. AMS, FG and CH designed the study, supervised the data collection, contributed to data analysis and commented on manuscript drafts. GDP and KC contributed to study design, assisted with accessing the field and commented on manuscript drafts. CO collected the data and commented on manuscript drafts. All authors reviewed and approved the final version of the manuscript.

Funding: This article presents independent research funded by the National Institute for Health Research (NIHR) under the Health Services and Delivery Research programme (project number 15/15/09). The views expressed in this publication are those of the authors and not necessarily those of the NIHR or the Department of Health and Social Care. KC is supported by an NIHR post-doctoral research fellowship.

Competing interests: CH is a member of the ReSPECT national working group and was involved in the evaluation of ReSPECT. GDP is a member of the ReSPECT national working group and held a leading role in the development of ReSPECT; however, GDP was not involved in data collection or analysis related to the present study. AS, FG, CH, KC and GDP received grants from the UK National Institute of Health Research during the study.

Ethics approval: The study received ethics approval from the NRES Committee, West Midlands – Coventry and Warwickshire (REC reference: 17/WM/0134).

Data sharing statement: Although the qualitative data in this study have been pseudonimised, it is possible that with access to raw data individuals might be identifiable. The data are not suitable for sharing beyond what is contained within the manuscript. Further information can be obtained from the corresponding author.

For peer review only



For peer review only - http://bmjopen.bmj.com/site/about/guidelines.xhtml

# Reporting checklist for qualitative study.

Based on the SRQR guidelines.

## Instructions to authors

Complete this checklist by entering the page numbers from your manuscript where readers will find each of the items listed below.

Your article may not currently address all the items on the checklist. Please modify your text to include the missing information. If you are certain that an item does not apply, please write "n/a" and provide a short explanation.

Upload your completed checklist as an extra file when you submit to a journal.

In your methods section, say that you used the SRQRreporting guidelines, and cite them as:

O'Brien BC, Harris IB, Beckman TJ, Reed DA, Cook DA. Standards for reporting qualitative research: a synthesis of recommendations. Acad Med. 2014;89(9):1245-1251.

| | | Reporting Item | Page Number |
|---|---|---|---|
| **Title** | | | |
| | #1 | Concise description of the nature and topic of the study identifying the study as qualitative or indicating the approach (e.g. ethnography, grounded theory) or data collection methods (e.g. interview, focus group) is recommended | 1 |

For peer review only

## Abstract

| | | | |
|---|---|---|---|
| | #2 | Summary of the key elements of the study using the abstract format of the intended publication; typically includes background, purpose, methods, results and conclusions | 1 |

## Introduction

| | | | |
|---|---|---|---|
| Problem formulation | #3 | Description and signifcance of the problem / phenomenon studied: review of relevant theory and empirical work; problem statement | 3 |
| Purpose or research question | #4 | Purpose of the study and specific objectives or questions | 3-4 |

## Methods

| | | | |
|---|---|---|---|
| Qualitative approach and research paradigm | #5 | Qualitative approach (e.g. ethnography, grounded theory, case study, phenomenolgy, narrative research) and guiding theory if appropriate; identifying the research paradigm (e.g. postpositivist, constructivist / interpretivist) is also recommended; rationale. The rationale should briefly discuss the justification for choosing that theory, approach, method or technique rather than other options available; the assumptions and limitations implicit in those choices and how those choices influence study conclusions and transferability. As appropriate the rationale for several items might be | 4 |

For peer review only

| | | | |
|---|---|---|---|
| | | discussed together. | |
| Researcher characteristics and reflexivity | #6 | Researchers' characteristics that may influence the research, including personal attributes, qualifications / experience, relationship with participants, assumptions and / or presuppositions; potential or actual interaction between researchers' characteristics and the research questions, approach, methods, results and / or transferability | 4 |
| Context | #7 | Setting / site and salient contextual factors; rationale | 4 |
| Sampling strategy | #8 | How and why research participants, documents, or events were selected; criteria for deciding when no further sampling was necessary (e.g. sampling saturation); rationale | 4 |
| Ethical issues pertaining to human subjects | #9 | Documentation of approval by an appropriate ethics review board and participant consent, or explanation for lack thereof; other confidentiality and data security issues | 19 |
| Data collection methods | #10 | Types of data collected; details of data collection procedures including (as appropriate) start and stop dates of data collection and analysis, iterative process, triangulation of sources / methods, and modification of procedures in response to evolving study findings; rationale | 4 |
| Data collection | #11 | Description of instruments (e.g. interview guides, | 4 |

For peer review only

| | | | |
|---|---|---|---|
| instruments and technologies | | questionnaires) and devices (e.g. audio recorders) used for data collection; if / how the instruments(s) changed over the course of the study | |
| Units of study | #12 | Number and relevant characteristics of participants, documents, or events included in the study; level of participation (could be reported in results) | 4 |
| Data processing | #13 | Methods for processing data prior to and during analysis, including transcription, data entry, data management and security, verification of data integrity, data coding, and anonymisation / deidentification of excerpts | 4-5 |
| Data analysis | #14 | Process by which inferences, themes, etc. were identified and developed, including the researchers involved in data analysis; usually references a specific paradigm or approach; rationale | 4-5 |
| Techniques to enhance trustworthiness | #15 | Techniques to enhance trustworthiness and credibility of data analysis (e.g. member checking, audit trail, triangulation); rationale | 4-5 |
| **Results/findings** | | | |
| Syntheses and interpretation | #16 | Main findings (e.g. interpretations, inferences, and themes); might include development of a theory or model, or integration with prior research or theory | 5-14 |
| Links to empirical data | #17 | Evidence (e.g. quotes, field notes, text excerpts, photographs) to substantiate analytic findings | 5-14 |

For peer review only

## Discussion

| | | | |
|---|---|---|---|
| Intergration with prior work, implications, transferability and contribution(s) to the field | #18 | Short summary of main findings; explanation of how findings and conclusions connect to, support, elaborate on, or challenge conclusions of earlier scholarship; discussion of scope of application / generalizability; identification of unique contributions(s) to scholarship in a discipline or field | 14-16 |
| Limitations | #19 | Trustworthiness and limitations of findings | 15-16 |

## Other

| | | | |
|---|---|---|---|
| Conflicts of interest | #20 | Potential sources of influence of perceived influence on study conduct and conclusions; how these were managed | 19 |
| Funding | #21 | Sources of funding and other support; role of funders in data collection, interpretation and reporting | 19 |

The SRQR checklist is distributed with permission of Wolters Kluwer © 2014 by the Association of American Medical Colleges. This checklist was completed on 13. May 2019 using https://www.goodreports.org/, a tool made by the EQUATOR Network in collaboration with Penelope.ai

For peer review only
